# Requirement of PML SUMO Interacting Motif for RNF4- or Arsenic Trioxide-Induced Degradation of Nuclear PML Isoforms

**DOI:** 10.1371/journal.pone.0044949

**Published:** 2012-09-18

**Authors:** Mohamed Ali Maroui, Sabrina Kheddache-Atmane, Faten El Asmi, Laurent Dianoux, Muriel Aubry, Mounira K. Chelbi-Alix

**Affiliations:** 1 CNRS, FRE 3235, Université Paris Descartes, Paris, France; 2 Département de Biochimie, Université de Montréal, Montréal, Canada; University of Virginia, United States of America

## Abstract

PML, the organizer of nuclear bodies (NBs), is expressed in several isoforms designated PMLI to VII which differ in their C-terminal region due to alternative splicing of a single gene. This variability is important for the function of the different PML isoforms. PML NB formation requires the covalent linkage of SUMO to PML. Arsenic trioxide (As_2_O_3_) enhances PML SUMOylation leading to an increase in PML NB size and promotes its interaction with RNF4, a poly-SUMO-dependent ubiquitin E3 ligase responsible for proteasome-mediated PML degradation. Furthermore, the presence of a *bona fide* SUMO Interacting Motif (SIM) within the C-terminal region of PML seems to be required for recruitment of other SUMOylated proteins within PML NBs. This motif is present in all PML isoforms, except in the nuclear PMLVI and in the cytoplasmic PMLVII. Using a bioluminescence resonance energy transfer (BRET) assay in living cells, we found that As_2_O_3_ enhanced the SUMOylation and interaction with RNF4 of nuclear PML isoforms (I to VI). In addition, among the nuclear PML isoforms, only the one lacking the SIM sequence, PMLVI, was resistant to As_2_O_3_-induced PML degradation. Similarly, mutation of the SIM in PMLIII abrogated its sensitivity to As_2_O_3_-induced degradation. PMLVI and PMLIII-SIM mutant still interacted with RNF4. However, their resistance to the degradation process was due to their inability to be polyubiquitinated and to recruit efficiently the 20S core and the β regulatory subunit of the 11S complex of the proteasome in PML NBs. Such resistance of PMLVI to As_2_O_3_-induced degradation was alleviated by overexpression of RNF4. Our results demonstrate that the SIM of PML is dispensable for PML SUMOylation and interaction with RNF4 but is required for efficient PML ubiquitination, recruitment of proteasome components within NBs and proteasome-dependent degradation of PML in response to As_2_O_3_.

## Introduction

Promyelocytic Leukemia (PML) protein (also named TRIM19 for Tripartite Motif 19) is the organizer of nuclear bodies (NBs) [1], which are small nuclear-matrix structures that exist in almost all mammalian cells. In response to diverse stimuli, PML NBs recruit a growing number of proteins implicated in different cellular processes such as oncogenesis, DNA damage response, apoptosis, senescence, protein degradation and antiviral defense [2]–[10]. Interestingly, based on databases analysis, PML partners were recently brought together in a comprehensive network containing 166 proteins [11]. A common known feature between the proteins found in PML NBs is their capacity to be SUMOylated. According to the current literature, almost 40% of PML partners have been confirmed to be SUMOylated. This suggests that PML NBs are enriched sites for SUMOylated proteins [11] and may function as nuclear SUMOylation hotspots [9], [12], [13].

Like many other proteins in PML NBs, PML is SUMOylated by SUMO1, SUMO2 and SUMO3 on three of its lysine residues (K65, K160, K490) [14]. The factors involved in the regulation of PML SUMOylation have not been clearly defined. However, the therapeutic agent, arsenic trioxide (As_2_O_3_) is known to enhance PML SUMOylation [15], a modification which has important consequences on PML functions. SUMOylation affects PML cellular localization, stability and ability to interact with other partners. SUMOylation of PML is critical for the NB formation since a PML mutant that cannot be SUMOylated (PMLIII-3KR mutated in the lysine sites K65, K160, K490) shows a nuclear speckle pattern [5], [16] but has a defect in recruiting other NB-associated partners [5].

In the nucleus, most of PML is expressed in the diffuse nuclear fraction of the nucleoplasm and only a small fraction is found in the matrix-associated NBs [6], [7], [17]. The transfer of PML from the nucleoplasm to NBs depends on PML post-translational modifications [6], [7], [17]. In response to As_2_O_3_, PML is phosphorylated through the mitogen-activated protein kinase MAPK pathway [18] leading to the transfer of PML from the nucleoplasm to the nuclear matrix and to the increase of PML SUMOylation and NB size. Interestingly, it has been shown that As_2_O_3_ binds directly to cysteine residues in zinc fingers located within PML RING domain replacing the zinc ion. The substitution of zinc by arsenic induces conformational changes of PML leading to PML oligomerization and increased interaction with the SUMO-conjugating enzyme Ubc9 [19]. Subsequently, As_2_O_3_ stimulates the formation of higher molecular weight poly-SUMO chains onto PML (poly-SUMOylation) [10], [20].

Poly-SUMOylated PML recruits a poly-SUMO-specific ubiquitin E3 ligase, namely the Really interesting New gene (RING) Finger protein 4 (RNF4; also known as SNURF) [20], as well as ubiquitin and proteasome components onto PML NBs leading to PML degradation [4], [10]. RNF4 was initially described as a SUMO-targeted ubiquitin ligase (STUbL) [21]. This RING finger protein [22] colocalizes with SUMO1 in PML NBs [23] and can also function as a co-activator of the androgen receptor [24]. In addition to the demonstrated role of RNF4 in the degradation of SUMOylated PML, PIAS1 (Protein Inhibitor of Activacted Stat1) was recently identified as a SUMO E3 ligase promoting PML SUMOylation that is required for As_2_O_3_-dependent PML degradation [25].

Several PML isoforms designated PMLI to PMLVII [26], are generated by alternative splicing from a single gene [2], [9], [27]. They share the N-terminal region (exons 1–3), which encodes the RBCC motif, whereas they differ in their C-terminal region due to alternative splicing of exons 4 to 9. The RBCC motif, which harbors a RING finger, two B-boxes and an α-helical Coiled-coil domain, is essential for PML homodimerization and interaction with specific proteins as well as for PML NB formation [26]. The variability of the C-terminal part of PML isoforms is important for the recruitment of specific interacting partners of PML [2], [9], [28], [29]. Several motifs have been identified in the C-terminus of PML: a nuclear localization signal (NLS) found in all nuclear PML isoforms (PMLI to VI in exon 6 at position 476–490), a nuclear exclusion signal (NES) found only in PMLI (at position 704–713) consistent with the nuclear and cytoplasmic distribution of this isoform [30], [31] and a SUMO Interacting Motif (SIM) only present in PMLI to PMLV (VVVI encoded by exon 7a at position 556–559) [32], [33]. Previous studies assessing PML SUMOylation and degradation in response to As_2_O_3_ have been performed mostly with PMLIII isoform [5], [10], [15]–[17], [34] and very little is known about the fate of other PML isoforms upon As_2_O_3_ treatment. PML SIM was proposed to mediate non-covalent interactions with other SUMOylated proteins and to promote their recruitment in PML NBs [32], [33]. The recent finding that PMLVI isoform, which does not contain the SIM, is still able to form NBs in PML^−/−^ cells [35] suggests that this domain is not essential for NB formation. Whether this domain is required for PML stability is not established.

In this study, we took advantage of previous development of bioluminescence resonance energy transfer (BRET) assays to detect simultaneously PML interaction with protein partners and As_2_O_3_-induced PML SUMOylation as well as degradation in living cells [16], [36]. We proved that BRET can be used to quantify both covalent and non-covalent interaction of SUMO with PML [16]. In brief, BRET monitors the interaction between a protein fused to a luciferase and a protein fused to yellow or green fluorescent protein (YFP or GFP). It is a proximity-based assay requiring that the donor of energy (luciferase fusion) and the acceptor (YFP or GFP fusion) are within 50 to 100 A° for an efficient energy transfer upon addition of a luciferase substrate [37], [38].

In this report, we analyzed the SUMOylation and degradation of the six nuclear PML isoforms (PMLI to VI) and the cytoplasmic PMLVII. We show that As_2_O_3_ enhanced the conjugation to SUMO and the interaction with RNF4 of all nuclear PML isoforms. As_2_O_3_ also induced the degradation of PMLI to PMLV. However, although PMLVI (lacking the SIM) or PMLIII mutated in the SIM interacted with RNF4, they were resistant to this process as they failed in response to As_2_O_3_ to be ubiquitinated and to recruit efficiently the 20S core and the β regulatory subunit of the 11S proteasome in PML NBs. The cytoplasmic PMLVII was neither SUMOylated nor degraded and consistently did not interact with RNF4. Interestingly, while the presence of a SIM was essential for As_2_O_3_-induced PML degradation, overexpression of RNF4 partially by-passed this requirement.

## Results

### SUMOylation of PML Isoforms

**Figure 1:**
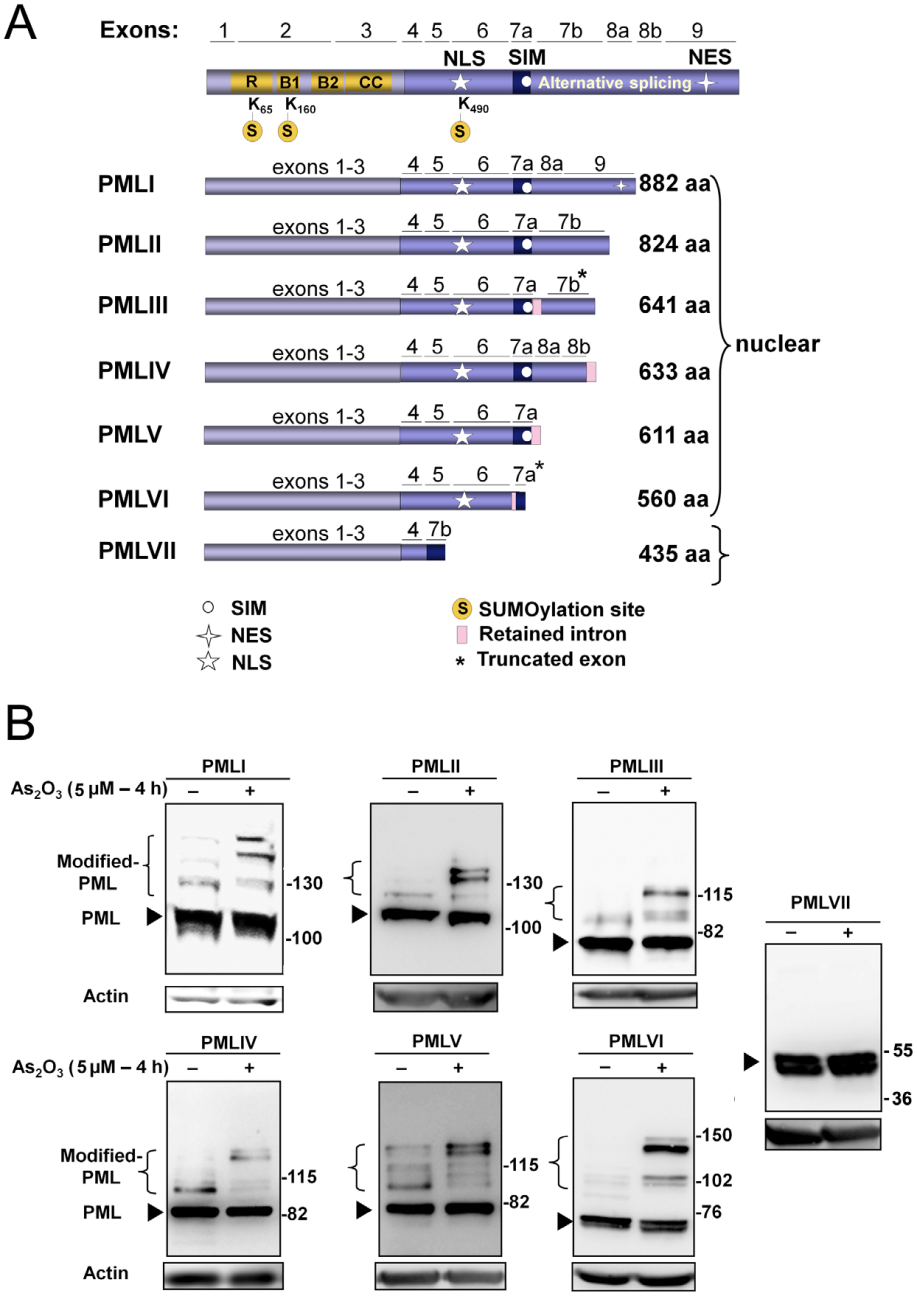
Increase in the SUMOylation of nuclear PML isoforms in response to As_2_O_3_ . (A) Schematic representation of the domain structure of PML isoforms. In addition to the seven PML isoforms (PMLI to PMLVII), a further division into sub-groups a/b/c reflects the alternative splicing of exons 4, 5 and 6 (“a” represents isoforms without exon 5, “b” isoforms without exon 5 and 6 and “c” isoforms without exon 4, 5 and 6) [26]. All PML isoforms share a common N-terminus but differ in their C-termini due to the alternative splicing of exons 4 to 9. Protein domains of PML include the RING finger (R), the B1 and B2 boxes, the Coiled-coil (CC) motif, the nuclear localization domain (NLS), the nuclear exclusion signal (NES), the three SUMOylation sites (K65, K160, K490) and the SUMO Interacting Motif (SIM). (B) U373MG cells stably expressing individual PML isoform (PMLI to VII) were treated in the presence or absence of 5 μM of As_2_O_3_ for 4 h. The cell extracts were analyzed by Western blot with anti-PML or anti-actin antibodies. The unmodified PML isoforms are indicated by arrowheads and the modified PML species by brackets.

Seven isoforms of PML generated by alternative splicing have been described (Fig. 1A) [26]. Among PML isoforms, six are nuclear (PMLI to VI) with PMLI being able to shuttle between the nucleus and the cytoplasm [31] and one is exclusively cytoplasmic (PMLVII). Previous studies have demonstrated that PMLIII is SUMOylated and that its SUMOylation is increased in response to As_2_O_3_ [5], [15], [16]. In order to determine whether all isoforms of PML are SUMOylated, we have stably expressed each of the PML isoforms (PMLI to VII) in U373MG cells [39] and compared their SUMOylation upon stimulation by As_2_O_3_. As previously shown with stably expressed PMLIII [5], [15], [16], an increase in the SUMOylation of all nuclear PML isoforms (PMLI to VI) was observed upon a short exposure to As_2_O_3_ (4 h) (Fig. 1B). This was evidenced on Western blots by an increase in the intensity of the bands of higher molecular weights (modified PML) migrating above the unmodified PML. The fact that PMLVI missing a SIM sequence was highly SUMOylated suggests that the SIM found in the other nuclear PML isoforms is not required for As_2_O_3_-induced PML SUMOylation. In contrast, no bands of higher molecular weights were observed for PMLVII in the presence or absence of As_2_O_3_, indicating that this cytoplasmic isoform was not SUMOylated.

**Figure 2:**
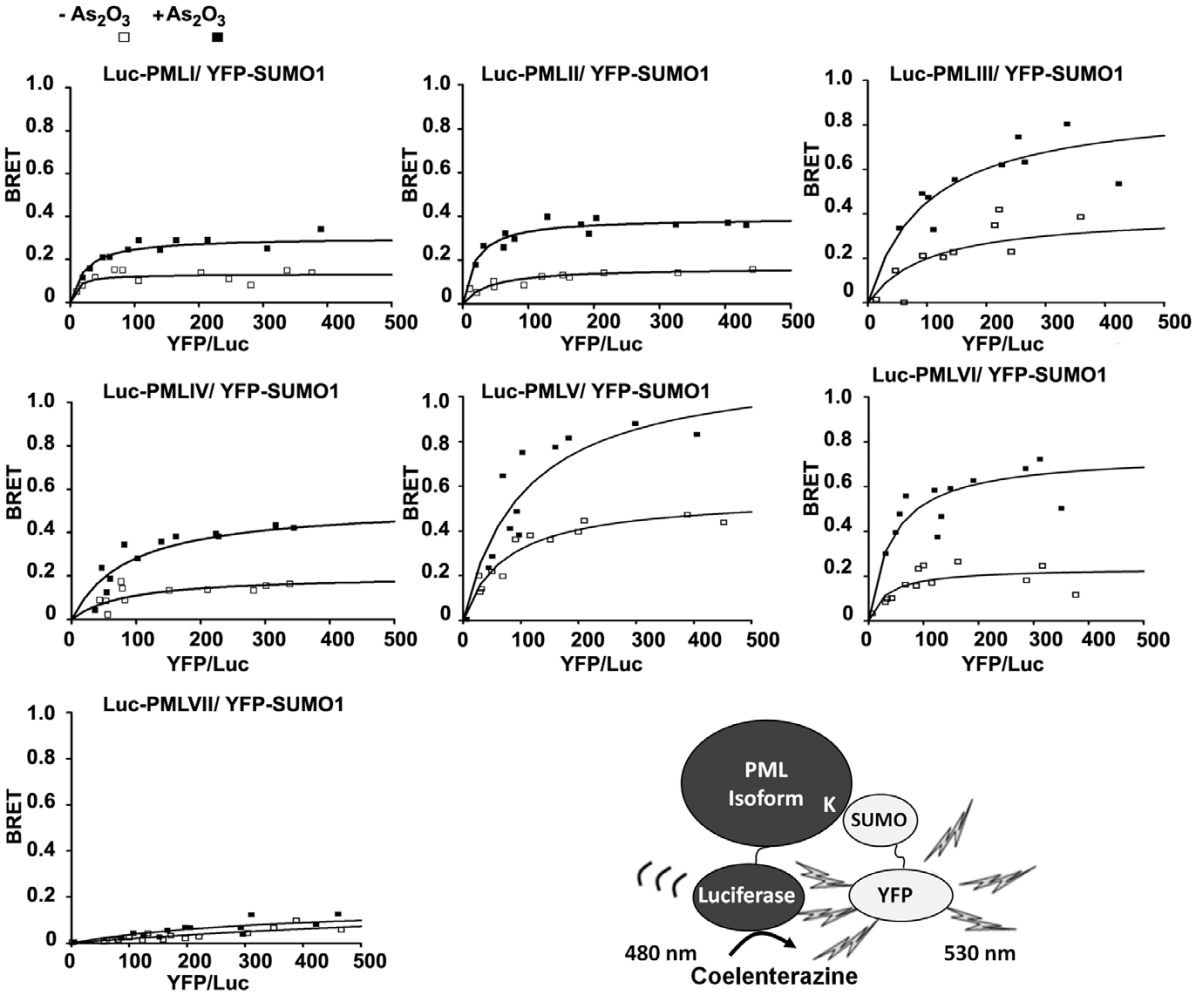
Detection by BRET of the increase in the SUMOylation of nuclear PML isoforms by SUMO1 in response to As_2_O_3_ in living cells. HEK293T cells, transiently transfected with a fixed amount of a Luc-PML fusion (PMLI to VII) and increasing amounts of YFP-SUMO1, were treated in the presence or absence of As_2_O_3_ (5 µM, 4 h) and used for BRET titration assays. BRET saturation curves are presented for each PML isoform in the absence (open square) or presence of As_2_O_3_ (closed square).The BRET donor of energy (luciferase fused to a PML isoform) and acceptor of energy (YFP fused to SUMO) are illustrated; upon addition of the membrane permeant luciferase substrate (coelenterazine Deep Blue), the bioluminescent signal resulting from the degradation of the substrate (emission 480 nm) is transferred to the YFP acceptor when the acceptor is within close proximity (50–100A°). The transferred energy results in a fluorescent signal emitted by the YFP acceptor (emission 530 nm). The BRET signal corresponds to the acceptor fluorescence/donor bioluminescence ratio (y axis) and is plotted as a function of the YFP/Luc fusion protein expression (x axis). ‘K’ represents a lysine to which SUMO can be covalently coupled.

The enhanced SUMOylation of all nuclear PML isoforms in the presence of As_2_O_3_ was also demonstrated in living cells by BRET (Fig. 2). Since the various PML isoforms have a different C-terminal region, they were tagged at their common N-terminus with *Renilla* luciferase (Luc-PML). SUMO1, 2 and 3 paralogs were also tagged at their N-terminus with YFP. HEK293T cells were co-transfected with a constant amount of each individual Luc-PML isoform construct and either a fixed amount (dose response) or increasing amount (BRET titration) of YFP-SUMO1, 2 or 3 construct. In each case, BRET was quantified by measuring the ratio of light emitted by the YFP acceptor and the luciferase donor upon addition of the membrane permeable luciferase substrate, coelenterazine. The BRET ratio was plotted as a function of the YFP/Luc fusion protein expression to take into account the potential variations in the expression of individual constructs. A significant increase in the BRET signal was obtained with all nuclear PML isoforms (PMLI to VI) in response to a short treatment (4 h) with various doses of As_2_O_3_ for each SUMO paralog (Fig. S1). An enhanced BRET signal was also obtained for all nuclear PML isoforms (PMLI-VI) at a fixed dose of As_2_O_3_ (5 µM for 4 h) using a constant amount of transiently transfected PML in the presence of increasing concentrations of either SUMO1 (Fig. 2), SUMO2 or SUMO3 (Figs. S2A and S2B) (BRET saturation curves). As previously demonstrated for PMLIII [16], this AO_3_-enhanced BRET signal observed in the dose response and titration assay saturation curves, is indicative of an increase in PML SUMOylation. In contrast, no significant change in the BRET signal was observed in the presence of As_2_O_3_ with the cytoplamic PMLVII (Fig. 2 and Figs. S1, S2A and S2B). Noticeably, a linear non-specific BRET signal resulting from random collision of Luc-PMLVII and YFP-SUMO (‘by stander BRET’) [40] was observed in the titration assays in both the presence and absence of As_2_O_3_ (Fig. 2 and Figs. S2A and S2B). This was contrasting with the BRET signal observed with PMLI to VI that was increasing as a hyperbolic function and was indicative of specific interaction [40]. Thus, Western blot analysis (Fig. 1B) and *in vivo* BRET data (Fig. 2 and Figs. S1, S2B and S2A) indicated that, in contrast to the nuclear isoforms, the cytoplasmic PMLVII was not SUMOylated by any of the SUMO parologs. As controls for the BRET assay, the localization and expression of all tagged PML isoforms in tranfected HEK293T cells were verified. PMLI to PMLVI presented the characteristic dots within PML NBs and as expected, PMLVII was found in the cytoplasm (Fig. S3A and data not shown). Western blots confirmed that all PML isoforms migrated at the expected molecular weight (Fig. S3B).

### Role of the SIM in the Degradation of PML Isoforms upon As_2_O_3_ Treatment

**Figure 3:**
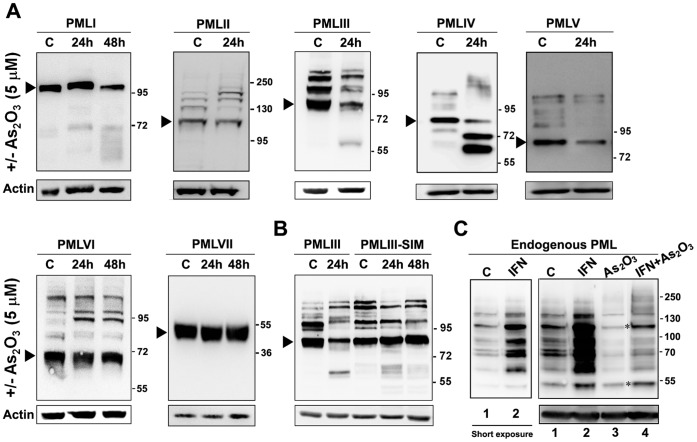
Differential degradation of PML isoforms in response to As_2_O_3_. (A) U373MG cells stably expressing each PML isoform were untreated (C) or treated with 5 µM of As_2_O_3_ for the indicated time (24 h or 48 h). (B) U373MG cells stably expressing PMLVI, PMLVII or PMLIII-SIM were untreated (C) or treated from 24 to 48 h with 5 μM of As_2_O_3_. The different cell extracts were analyzed by Western blot with anti-PML or anti-actin antibodies. (C) U373MG cells were untreated (C, lane 1) or treated with 1000 units/ml of IFNγ (lanes 2 and 4) to increase the expression of the endogenous forms of PML. One day later, As_2_O_3_ was added at the concentration of 5 μM (lanes 3 and 4) for 24 h. In A, B and C, cell extracts were analyzed by Western blot with anti-PML (top) or anti-actin antibodies (bottom). Unmodified PML isoforms are indicated by arrowheads and PML forms resistant to As_2_O_3_-induced degradation in IFN-treated cells by stars.

Previous studies have demonstrated that As_2_O_3_ stimulates the SUMOylation of stably expressed PMLIII when used for short periods and leads to its degradation if used for prolonged periods [4], [5], [10], [15]–[17]. This degradation process requires the C-terminal region of PMLIII [17]. In order to better understand the determinants of the As_2_O_3_-induced PML degradation, we compared the degradation of the seven PML isoforms, which differ in their C-terminal region. U373MG cells, stably expressing each of the different PML isoforms (PMLI to VII), were treated with As_2_O_3_ for a minimum of 24 h. The cell extracts were analyzed by Western blot to follow the kinetics of SUMOylation and expression of PML isoforms (Fig. 3A). The amount of PML decreased within 24 to 48 h of As_2_O_3_ treatment for all nuclear PML isoforms except for PMLVI. Indeed, PMLVI levels stayed relatively constant after a prolonged As_2_O_3_ treatment when compared to that of untreated cells. Although all nuclear PML isoforms include a caspase site (spanning Asp 522) [41], cleavage products only accumulated with PMLIV and to a lesser extend with PMLI and PMLIII. Finally, the cytoplasmic PMLVII, that was not SUMOylated upon treatment (Fig. 1B), remained resistant to As_2_O_3_-induced degradation (Fig. 3B).

Since PMLVI was resistant to As_2_O_3_-induced degradation and was the only nuclear isoform lacking a SIM, we directly addressed the role of the SIM in PML degradation. For that purpose, we compared the fate of PMLIII with that of its SIM mutated version (PMLIII-SIM) in response to a prolonged As_2_O_3_ treatment. Whereas PMLIII degradation was observed within 24 h, the PMLIII-SIM was resistant to degradation up to 48 h (Fig. 3B). This demonstrated the requirement of the SIM in the degradation process. As shown for PMLVI (Fig.1B and Fig. 3A), PMLIII-SIM was also SUMOylated in response to As_2_O_3_ (Fig. 3B and Fig. S3C), suggesting that resistance to As_2_O_3_-induced degradation was not due to an impaired SUMOylation event. The degradation of PML in response to As_2_O_3_ was also analyzed in the context of endogenous PML. To facilitate the identification of endogenous PML species, we used interferon (IFN) to enhance the expression of PML isoforms [42]. The levels of many endogenous PML species were increased in response to IFN (Fig. 3C, lane 2) as previously described [42] whereas their expression levels were highly decreased following prolonged exposure to As_2_O_3_ (24 h) (Fig. 3C, lane 3). Interestingly, two main forms with apparent molecular weight of ∼ 55,000 and 120,000 were found to be resistant to As_2_O_3_ degradation when used alone or in combination with IFN (indicated by stars in Fig. 3C, lanes 3–4). Analysis of cytoplasmic and nuclear extracts from cells treated with IFN alone or IFN combined with As_2_O_3_ showed that the 55 kDa form was cytoplasmic suggesting that it correspond to PMLVII whereas the 120 kDa PML form was nuclear and could represent a SUMOylated form of PMLVI (Fig. S3D).

### Role of the SIM in RNF4-mediated Degradation of Nuclear PML Isoforms in the Presence or Absence of As_2_O_3_

As_2_O_3_ induced SUMO-dependent ubiquitin-mediated proteosomal PML degradation in stably transfected cells as these used in Fig. 3A [5], [16]. However, in transiently transfected cells, no significant change in PML level was observed with any PML isoform upon treatment with As_2_O_3_. The inability of As_2_O_3_ to degrade PML in transiently transfected cells could be due to the high level of transiently expressed PML compared to the endogenous components of the ubiquitin-mediated proteasomal pathway such as the RNF4, the poly-SUMO-specific ubiquitin ligase involved in the control of PML degradation [4], [10], [16]. To further understand the determinants involved in RNF4 and/or As_2_O_3_-induced PML degradation, we tested the effect of RNF4 on the expression of each PML isoform in transiently co-transfected cells in the presence or absence of As_2_O_3_.

**Figure 4:**
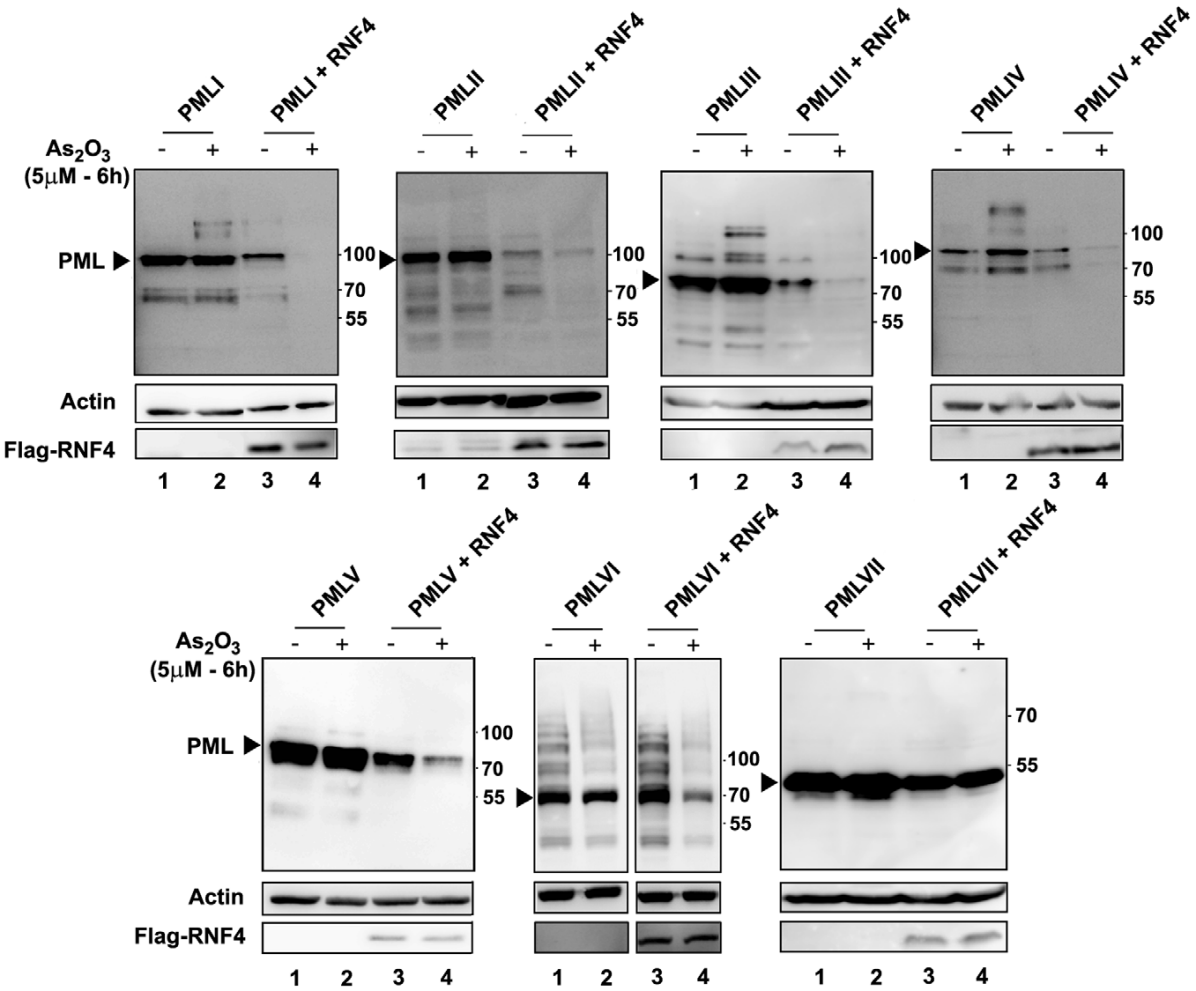
Degradation of PML isoforms in the presence of RNF4 and/or As_2_O_3_. U373MG cells were transiently co-transfected with each of the different PML isoforms (PMLI to VII) and RNF4 (ratio PML/RNF4∶1/1). One day post-transfection, cells were treated with 5 μM for 6 h. The different cell extracts were analyzed by Western blot with anti-PML, anti-Flag (Flag-RNF4) or anti-actin antibodies. Unmodified PML isoforms are indicated by arrowheads. For each PML isoform, samples in the presence (lanes 1–2) and in the absence of RNF4 (lanes 3–4) were loaded on the same gel to allow comparison of the protein levels.

Previously, we and others demonstrated that exogenous RNF4 was sufficient to trigger a proteasome-dependent PMLIII degradation in transiently co-transfected cells [4], [10], [16]. Here, expression of RNF4 led to a decrease in the level of all nuclear PML isoforms except PMLVI in the absence of As_2_O_3_ (Fig. 4, compare lanes 1 and 3). Note that As_2_O_3_ alone did not change significantly the level of any PML isoform individually expressed in transiently transfected cells (lanes 2). However, the addition of As_2_O_3_ for a short period (6 h) to cells transfected with RNF4 resulted in a more dramatic decrease in the level of PMLI to PMLV isoforms. Strikingly, a degradation of PMLVI was also observed in the presence of both RNF4 and As_2_O_3_ (Fig. 4, lanes 4). Thus, PMLVI became partially sensitive to degradation following the combination of exogenous expression of RNF4 and exposure to As_2_O_3_. In contrast, the cytoplasmic PMLVII remained resistant to As_2_O_3_-induced degradation both in the presence and absence of RNF4.

### Interaction of RNF4 with SUMOylated PML Isoforms

**Figure 5:**
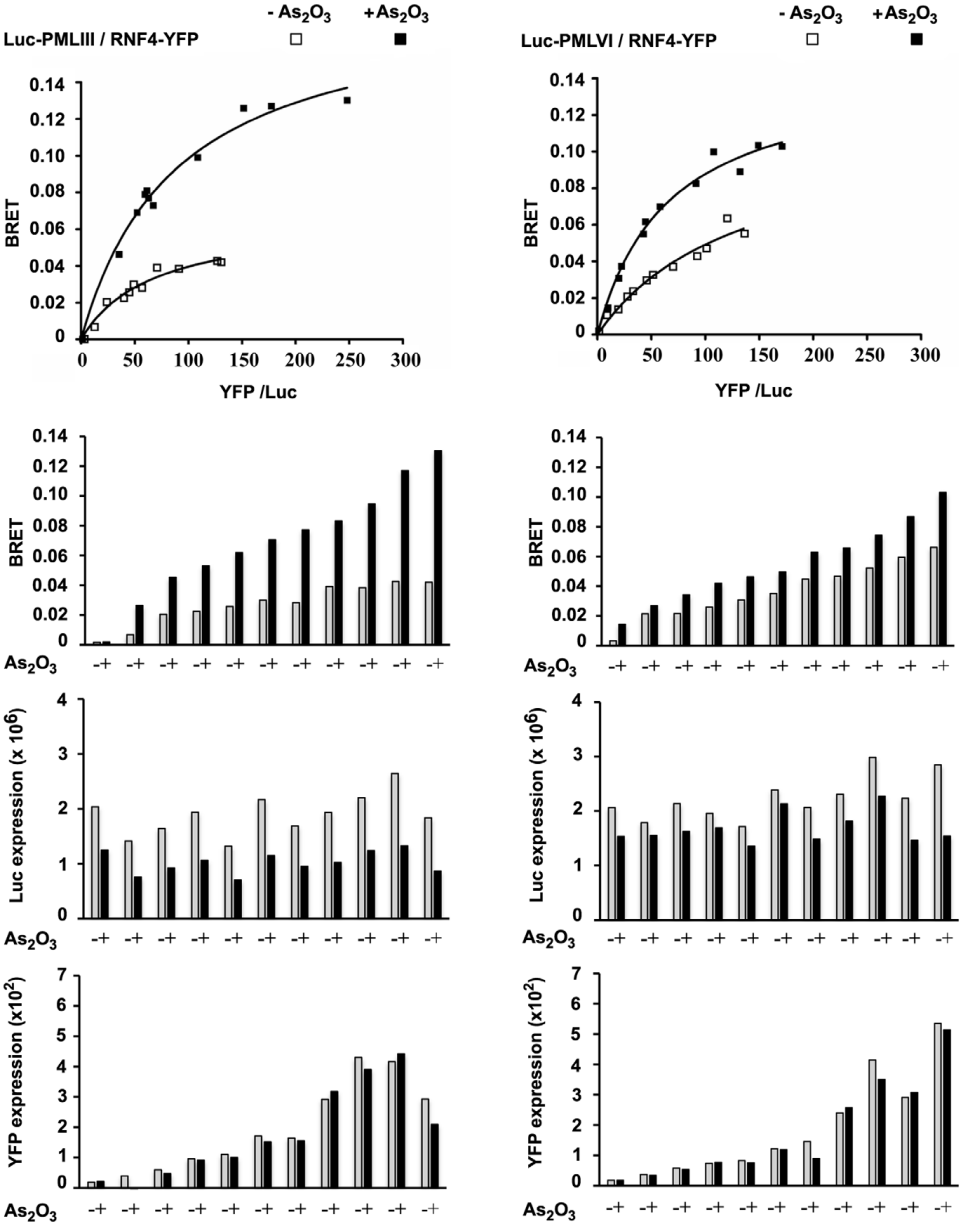
Interaction by BRET of RNF4 with SUMOylated PMLIII or PMLVI. HEK293T cells transiently transfected with a Luc-PML fusion (PMLIII or VI) and increasing amounts of RNF4-YFP, were untreated or treated with As_2_O_3_ (5 µM, 4 h) and used for BRET titration assays. BRET saturation curves are presented for each PML isoform in the absence (open square) or presence of As_2_O_3_ (closed square). Bar graphs are for presenting the BRET signal (BRET), the luciferase expression and YFP expression of individual untreated (gray bars) or treated (black bars) samples. A strong increase in the YFP/Luc expression ratios upon As_2_O_3_ treatment was observed for the BRET pair Luc-PMLIII/RNF4-YFP when compared to the YFP/Luc ratios obtained with the same untreated samples. This increase in the YFP/Luc expression ratios was accounted by a significant decrease in the level of Luc-PMLIII in the presence of As_2_O_3_ whereas the level of RNF4-YFP remained essentially unaffected by the treatment; this is evidenced by the bar graphs of the treated and untreated samples presented below the BRET curves. The shift toward higher YFP/Luc ratios resulting from Luc-PML degradation was much more limited for PMLVI than for the other nuclear PML isoforms such as PMLIII (this figure) and PMLI to PMLV (Figs. S4A and S4B). Noticeably, in response to As_2_O_3_, the Luc-PML reduced expression was not found to be dependent on the concentration of RNF4 added. Since this assay is conducted in conditions of overexpression of both PML and RNF4 in transiently transfected cells, we believe that this is possibly due to the fact that, in comparison with PML and RNF4, the ubiquitin and/or proteasomal machineries were limiting in the cells.

We have previously shown by BRET that PMLIII interacts with RNF4 in a SUMO-dependent manner since the SUMOylation deficient mutant, PMLIII-3KR, fails to interact with RNF4 [16]. To determine if the degradation of PML is dependent on the differential interaction of RNF4 with the various SUMOylated PML isoforms, we assessed these interactions by BRET in the presence or absence of As_2_O_3_. BRET saturation curves indicated that all nuclear PML isoforms presented an interaction with RNF4 that was enhanced by As_2_O_3_ (Fig. 5 and Figs. S4A, and S4B). Also, in the presence of As_2_O_3_, a clear shift towards higher YFP/Luc expression ratios was observed in the BRET curves with PMLI to PMLV (from 79 to 150% increase in the YFP/Luc ratio at the highest concentration of RNF4) (Fig. 5 and Figs. S4A and S4B) whereas this shift was quite limited with PMLVI (only 21% increase at the highest concentration of RNF4) (Fig. 5). As previously described for the PMLIII/RNF4 BRET pair [16], this shift towards higher YFP/Luc expression ratios was essentially due to i) a decrease in the level of Luc-PML consistent with PML degradation upon As_2_O_3_ treatment as shown in separate bar graphs of each individual untreated and treated samples (Fig. 5 and Figs. S4A and S4B) and ii) the fact that the BRET signal depends on the ratio of expression of the Luc donor fusion and YFP acceptor fusion proteins (plotted in saturation curves as a function of the protein expression ratio named YFP/Luc) (see a more detailed description in Fig. 5 legend). Thus, the BRET assay demonstrated that i) the interaction of RNF4 with all nuclear PML isoforms was reinforced in response to As_2_O_3_, and ii) unlike the SIM-containing PML isoforms, PMLVI was less sensitive to degradation induced by overexpression of RNF4 in combination with As_2_O_3_ treatment. In contrast, the cytoplasmic PMLVII did not interact with RNF4 since only a non-specific BRET signal was detected (Fig. S4C). Taken together, our results indicated that the differential sensitivity of PMLVI relative to all the other PML isoforms to As_2_O_3_-induced degradation did not depend on a defective interaction with RNF4.

### The SIM of PML is not Required for the Interaction with RNF4 but is Necessary for PML Ubiquitination in Response to As_2_O_3_

**Figure 6:**
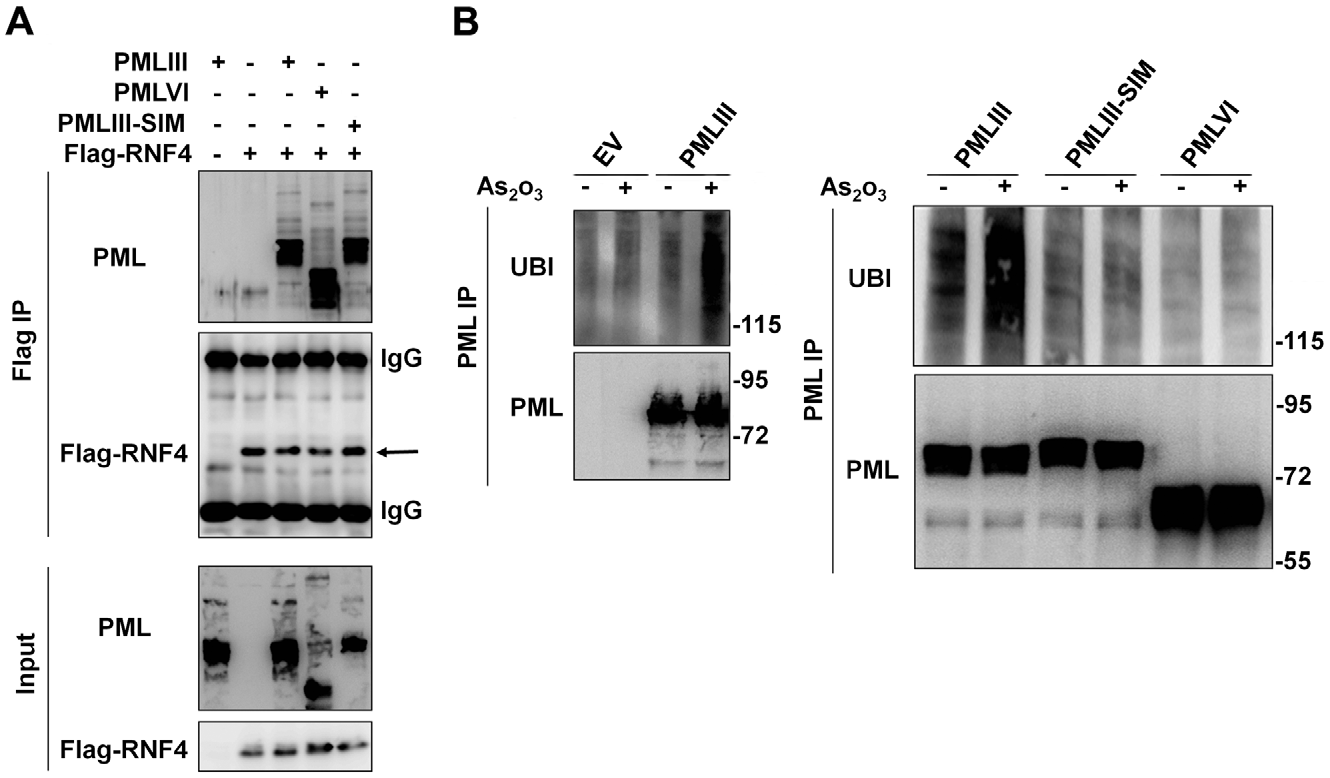
The SIM of PML is not required for the interaction with RNF4 but is necessary for PML ubiquitination in response to As_2_O_3_. (A) Co-immunoprecipitation of RNF4 and PML isoforms and mutant. HEK293 cells were co-tranfected with a construct encoding Flag-RNF4 and PMLIII, PMLVI or PMLIII-SIM. As controls, cells were transfected with PMLIII or RNF4 alone. Two days later, the different cell extracts were immunoprecipitated with an anti-Flag antibody as described in Materials and Methods and samples were analyzed by Western blot using anti-PML and anti-Flag antibodies. (B) Ubiquitination of PML in response to As_2_O_3_. PML^−/−^ MEFs were transiently transfected with the empty vector (EV) or PMLIII (left panel) or contructs encoding PMLIII, PMLIII-SIM or PMLVI (right panel). Two days later, they were treated with As_2_O_3_ for 1 h. The cell extracts were immunoprecipitated with rabbit anti-PML antibody and the immunoprecipitate was analyzed by Western blot using anti-ubiquitin and anti-PML antibodies.

Since BRET experiments indicated that PMLVI missing a SIM interacted with RNF4 (Fig. 5), we further confirmed this data by coimmunoprecipitation assays. Cell extracts from cells co-transfected with Flag-RNF4 and PMLIII, PMLVI or PMLIII-SIM constructs were used for co-immunoprecipitation. As seen by Western blot, the anti-Flag antibody co-immunoprecipitated PMLIII, PMLVI and PMLIII-SIM (Fig. 6A). As expected, such interactions were not observed with extracts from cells transfected with PMLIII or RNF4 alone. This further demonstrated that the SIM of PML was not required for the interaction with RNF4.

To further analyze why PML missing the SIM interacted with RNF4 but was still resistant to As_2_O_3_-induced degradation (Fig. 3), we tested whether this was due to a defect in its ubiquitination. For this purpose, PMLIII, PMLIII-SIM or PMLVI were transiently transfected in PML^−/−^ MEFs and cells were treated for 1 h with As_2_O_3_. Western blot analysis of immunoprecipitated PML (Fig. 6B) revealed that PMLIII, but not PMLIII-SIM or PMLVI, was polyubiquitinated in response to As_2_O_3_. Thus, resistance to As_2_O_3_-induced degradation of PMLVI and PMLIII-SIM mutant could be due to their inability to be efficiently ubiquitinated.

### Requirement of the SIM for the Recruitment of the 20S Core and the β Regulatory Subunit of the 11S Complex of the Proteasome to PML NBs in Response to As_2_O_3_

**Figure 7:**
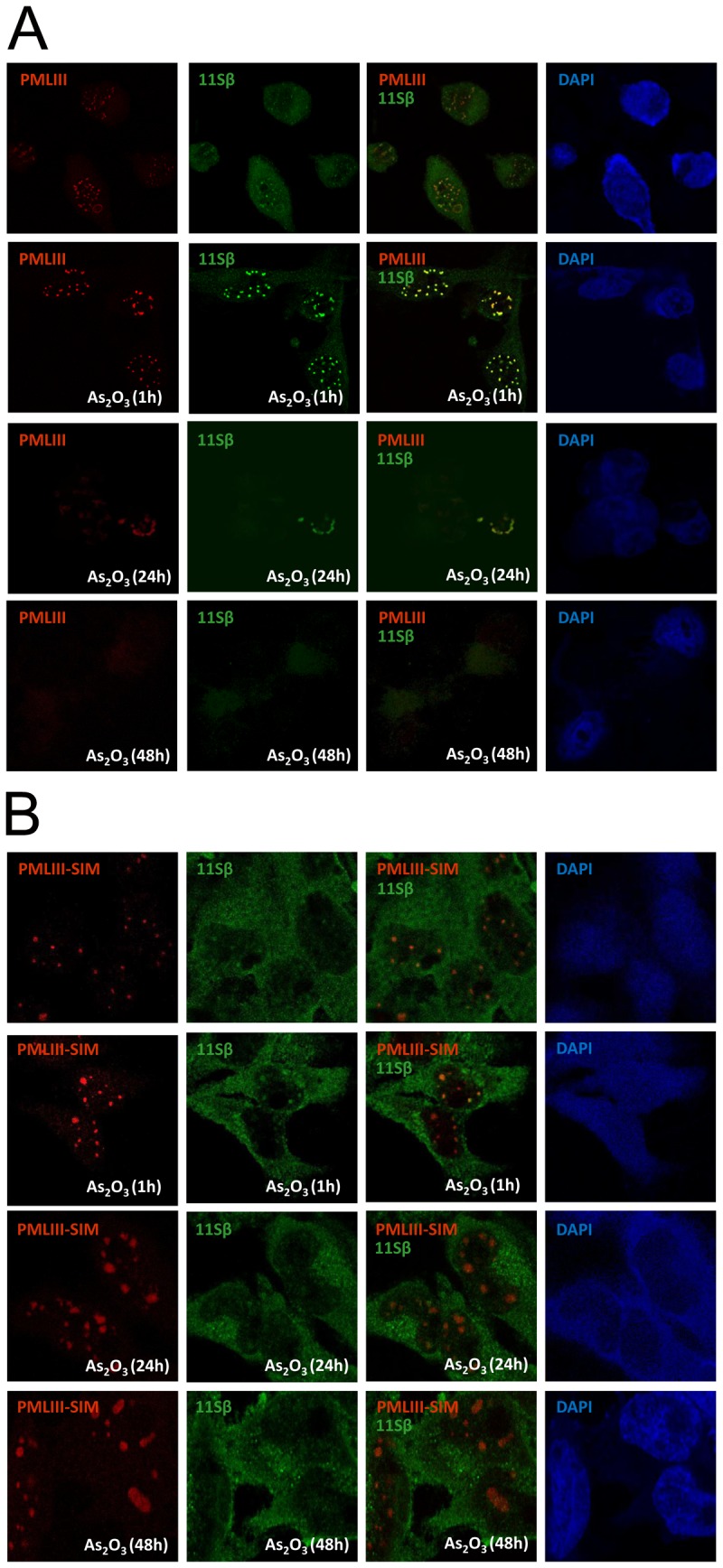
Requirement of the SIM for the recruitment of theβ regulatory subunit of the 11S proteasome to PML NBs in response to As_2_O_3_. Confocal immunofluorescence analysis of PML and of the β regulatory subunit of the 11S proteasome (11Sβ) were performed on U373MG cells stably expressing PMLIII (A) or PMLIII-SIM (B), treated or not with 5 μM of As_2_O_3_ for 1 h, 24 h or 48 h. PML and 11Sβ were detected with a mouse anti-PML and a rabbit anti-11Sβ antibodies followed by the corresponding anti-IgG antibody conjugated to Alexa 594 (red) and 488 (green), respectively. The merged images at 1 h of As_2_O_3_ treatment revealed in all cells a co-localization of endogenous 11Sβ with PMLIII but not with PMLIII-SIM mutant.

**Figure 8:**
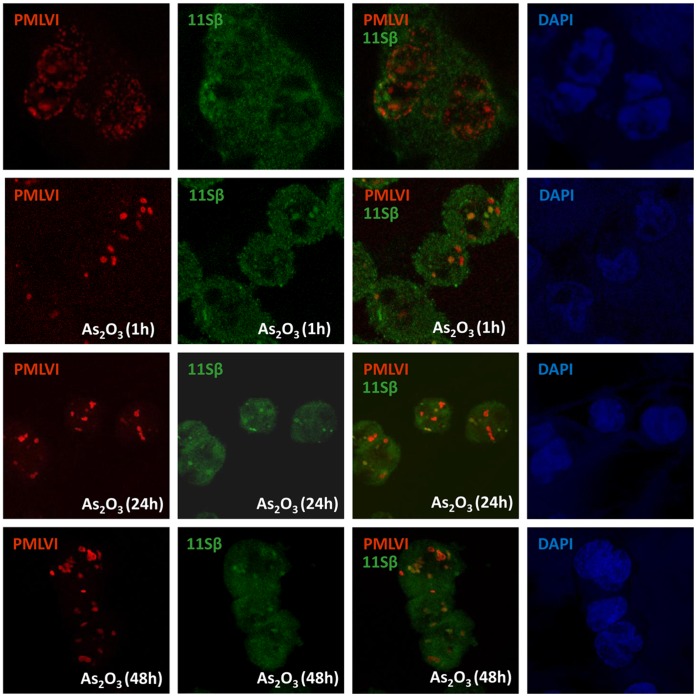
PMLVI was unable to recruit the β regulatory subunit of the 11S proteasome to PML NBs in response to As_2_O_3_. Confocal immunofluorescence analysis of PML and11Sβ were performed on U373MG cells stably expressing PMLVI treated or not with 5 μM of As_2_O_3_ for 1 h, 24 h or 48 h. PML and 11Sβ were detected with a mouse anti-PML and a rabbit anti-11Sβ antibodies followed by the corresponding anti-IgG antibody conjugated to Alexa 594 (red) and 488 (green), respectively.

**Figure 9:**
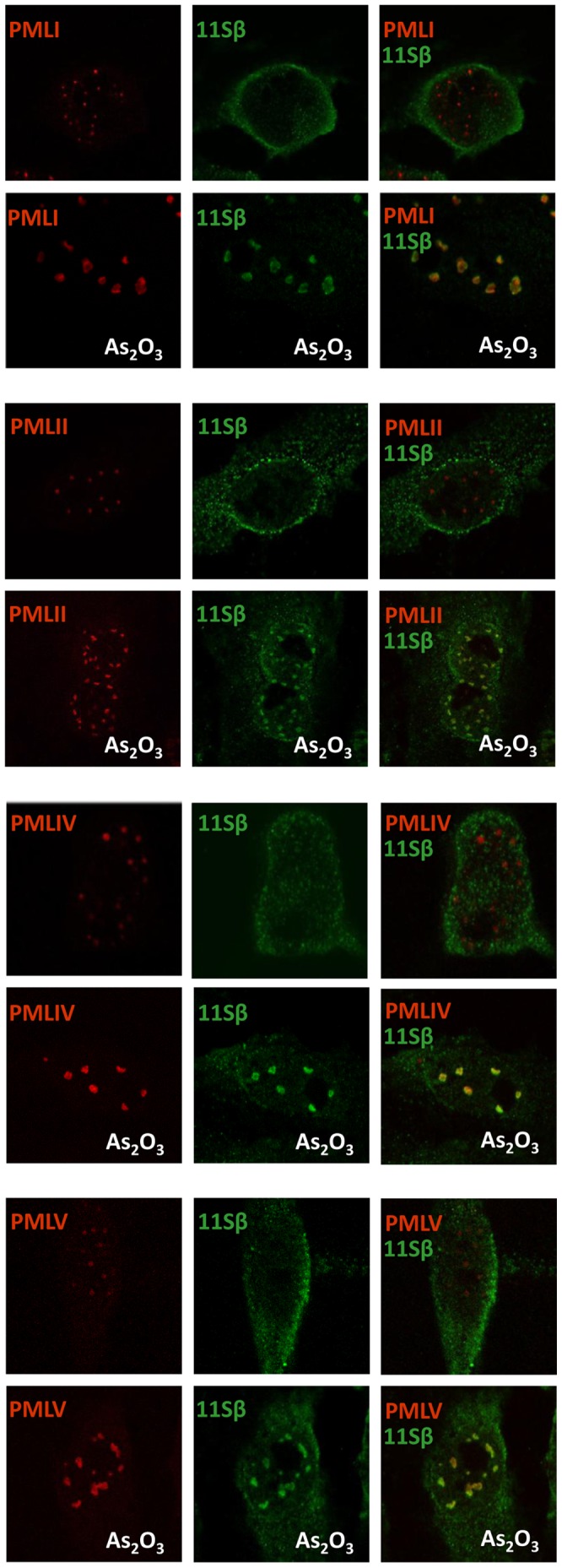
The β regulatory subunit of the 11S proteasome was recruited to PML NBs in response to As_2_O_3_ in cells stably expressing PMLI, PMLII, PMLIV and PMLV. Confocal immunofluorescence analysis of PML and 11Sβ were performed on U373MG cells stably expressing PMLI, PMLII, PMLIV or PMLV, treated or not with 5 μM of As_2_O_3_ for 1 h. PML and 11Sβ were detected with a mouse anti-PML and a rabbit anti-11Sβ antibodies followed by the corresponding anti-IgG antibody conjugated to Alexa 594 (red) and 488 (green), respectively. The merged images revealed co-localization of PML and endogenous 11Sβ for PMLI, PMLII, PMLIV and PMLV in the presence of As_2_O_3_ in nearly all cells.

As_2_O_3_-triggered degradation of PMLIII is associated to targeting of this isoform to the PML NBs and to the recruitment of the proteasome components to the PML NBs [5]. Since the SIM of PML was shown here to be required for RNF4-mediated and As_2_O_3_-induced PML degradation as well as for As_2_O_3_-induced ubiquitination, we tested its possible requirement for the recruitment of the 20S catalytic core and the β regulatory subunit of the 11S complex (11Sβ) of the proteasome in PML NBs. For this purpose, cells stably expressing PMLIII, PMLIII-SIM or PMLVI were treated in the presence or absence of As_2_O_3_ and submitted to double immunofluorescence staining for PML and either endogenous 20S core (Fig. S5) or endogenous 11Sβ (Figs. 7 and 8). It is known that PMLIII bodies became larger/brighter in cells treated for short period (1 h) with As_2_O_3_, due to the transfer of PML from the nucleoplasm to the nuclear matrix and to PML SUMOylation [5], [17]. Thus, as expected, an increase in the PML NB size was triggered by As_2_O_3_ with all nuclear PML isoforms as well as with PMLIII-SIM (Figs. 7, 8, 9 and S5). In the absence of As_2_O_3_, any or very few colocalization was observed between PMLIII and the proteasome components (Figs. 7, 8, 9 and S5). Following 1 h of As_2_O_3_ treatment, PMLIII colocalized with the 20S core (Fig. S5) or 11Sβ(Fig. 7) indicating a recruitment of the proteasome by PMLIII within NBs. Similar recruitment of proteasome components within PML NBs was obtained with all the other SIM-containing nuclear PML isoforms (PMLI, PMLII, PMLIV, PMLV) as shown using 11Sβ (Fig. 9). In contrast, only few or no colocalization of the proteasome was detected with PMLIII-SIM and PMLVI in response to As_2_O_3_ (Figs. 7, 8 and S5) demonstrating that PMLIII-SIM and PMLVI, which miss a functional SIM, failed to recruit efficiently proteasome components in PML NBs.

In addition, Kinetics immunofluorescence studies (1 h, 24 h and 48 h of As_2_O_3_ treatment) performed with PMLIII, PMLIII-SIM and PMLVI (Figs. 7 and 8) demonstrated that PMLIII labeling decreased at 24 h and totally disappeared at 48 h whereas that of PMLIII-SIM mutant and PMLVI remained stable further confirming our results by Western blot (Fig. 3) that PMLIII-SIM and PMLVI were resistant to As_2_O_3_-induced degradation. Furthermore, PMLIII-SIM and PMLVI never recruited efficiently the 11Sβ during this whole Kinetics (Figs. 7 and 8).

Taken together, our results point to the requirement of the SIM of PML in As_2_O_3_-induced recruitment of proteasome components within the NBs and consecutive proteasome-dependent degradation of PML.

## Discussion

By binding SUMO proteins, the SIMs encoded in many nuclear proteins specify or strengthen the interaction with SUMOylated proteins. In nuclear PML isoforms, the SIM is present in PMLI to PMLV and is lacking in the nuclear PMLVI and in the cytoplasmic PMLVII. Here, we investigated the potential role of the SIM in the SUMOylation of the different PML isoforms, their interaction with RNF4 as well as their RNF4- and As_2_O_3_-induced degradation. The data presented provide a better understanding of the role of this motif in PML regulation. Altogether, our results suggest that upon As_2_O_3_ treatment, which triggers increased polySUMOylation of PML, i) the recruitment of the E3 ubiquitin ligase RNF4 by all the SUMOylated nuclear PML isoforms is enhanced, ii) the SIM of PML is dispensable for the basal and As_2_O_3_-enhanced interaction with RNF4, and iii) the SIM of PML is required for the efficient ubiquitination of PML and the targeting the 20S core and the β regulatory subunit of the 11S complex of the proteasome to PML NBs leading to As_2_O_3_-induced PML degradation. We also report that the overexpression of RNF4 solely induces the degradation of SIM-containing PML isoforms only whereas it alleviates the resistance of PML lacking the SIM to As_2_O_3-_induced degradation.

### The SIM of PML is Dispensable for PML SUMOylation in the Nucleus

Both our data obtained in living cells (BRET assays) and cell extracts (Western blots) indicate that all nuclear PML isoforms are conjugated to the three SUMO paralogs and that these modifications are enhanced by cell treatment with the therapeutic agent, As_2_O_3_. They also demonstrate that the SIM of PML is not required for PML SUMOylation as PMLVI lacking a SIM and PMLIII-SIM are efficiently SUMOylated. Similarly, the SIM of the Homeodomain-interacting protein kinase 2 (HIPK2) was shown to be dispensable for the modification of this kinase by SUMO1 [43]. At the opposite, it has been shown that the proper SUMOylation of other proteins such as Daxx or Bloom syndrome protein (BLM) necessitates the integrity of their SIM sequence [44], [45]. Indeed, the capacity of Daxx SIM to bind SUMO controls Daxx SUMOylation and function [44], [46], [47] and BLM SIM is required for preferential modification by SUMO2/3 [45].

As_2_O_3_ binds directly to cysteine residues in zinc fingers located within the RBCC domain of PML [19]. This results in PML RBCC-mediated oligomerization that leads to an increase in its interaction with SUMO-conjugating enzyme Ubc9 and to an enhanced PML SUMOylation and degradation. Our data demonstrate that the cytoplasmic PMLVII, which contains the RBCC motif, is however neither SUMOylated nor degraded in response to As_2_O_3_. This indicates that these processes of PML SUMOylation and regulation occur in the nucleus.

### The SIM of PML is Dispensable for Interaction with RNF4

Here, we observed by BRET that all nuclear PML isoforms, including PMLVI, interacted with RNF4 and that these interactions were strengthened in response to As_2_O_3_. Our results reveal that resistance of PMLVI to RNF4-mediated or As_2_O_3_-induced degradation is not due to a lack of interaction of this isoform with RNF4. As shown here, the BRET assay provides a method to simultaneously monitor SUMO-dependent PML/RNF4 interaction and PML degradation in the presence or absence of As_2_O_3_. Indeed, it provides a quantitative assessment of the interaction of the BRET protein partners and of their relative level of expression in the presence or absence of the drug. This represents a great advantage for comparing the interaction of RNF4 with the various PML isoforms, which have different sensitivity to RNF4- and As_2_O_3_-induced degradation.

We previously reported that the interaction of RNF4 with PML must occur indirectly via the SUMO moiety since no interaction was detected by BRET with a SUMOylation deficient PMLIII mutant [16]. This is consistent with the increased interaction of RNF4 with PML isoforms in response to As_2_O_3_, an agent known to induce poly-SUMOylation (as seen in Fig. 1B), and to the higher affinity of RNF4 for poly-SUMO chains [4], [10], [16]. In agreement, Fluorescence Resonance Energy Transfer (FRET) experiments indicate that RNF4 interacts with SUMO [48]. While detected by BRET, the SUMO-dependent interaction of PML with RNF4 was not detected in the absence of As_2_O_3_ by FRET. This is not due to a difference between the distances allowing interaction detection between FRET and BRET since the Forster distances are similar for the two techniques [49], [50]. Thus, it is most likely resulting from to the fact that BRET is more sensitive than FRET [49].

### The SIM of PML is Required for Efficient PML Ubiquitination, Recruitment of Proteasome Components within PML NBs and PML Degradation in Response to As_2_O_3_

In this report, based on the study of the various nuclear PML isoforms, we provide several lines of evidence that the SIM is required for the regulation of PML degradation induced by RNF4 or As_2_O_3_. First, As_2_O_3_ treatment sequentially leads to an increase in the SUMOylation of all nuclear PML isoforms and, on prolonged exposure, to the degradation of SIM-containing PML isoforms but not of PMLVI missing the SIM. Second, mutation of the SIM in PMLIII renders this isoform resistant to As_2_O_3_-induced degradation. Our finding is in agreement with a previous report showing that a C-terminal mutant of PMLIII, PMLIII-Stop504 (missing the SIM at the position 556–559), is resistant to the degradation in response to As_2_O_3_ [17]. Third, unlike the other SIM-containing PML isoforms, PMLVI is also resistant to the degradation mediated by exogenous RNF4. Interestingly, resistance of PMLVI to the degradation induced by either exogenous RNF4 or As_2_O_3_ is alleviated by a combination of RNF4 overexpression and As_2_O_3_ treatment. Thus, our results demonstrate that in the absence of a *bona fide* SIM in PML, the RNF4- or As_2_O_3_-induced degradation of PML is abolished, revealing that the SIM is important for efficient PML catabolism. Fourth, in response to As_2_O_3_, two key events ultimately leading to PML degradation are dependent on the presence of the SIM of PML. These are the ubiquitination of PML and the recruitment of proteasome components to PML NBs. Accordingly, the resistance to As_2_O_3_-induced degradation of PMLVI lacking a SIM and PMLIII-SIM mutant is attributed to their inability to be ubiquitinated and to recruit the 20S core and 11Sβ of the proteasome to PML NBs. Interestingly, PMLVI is very similar to PMLV (see Figure 1) but the latter include a SIM. Since PMLV has been shown to have the longest residence time within PML bodies and may serve as a scaffold for PML NBs [35], it would be interesting to determine the role of the SIM of PMLV in this process and to assess the As_2_O_3_-induced degradation and recruitment of the proteasome using a PMLV mutated in its SIM.

PML SIM has been suggested to participate in the nucleation of SUMOylated PML at NBs and the recruitment of SUMO-modified proteins [32]. For example, the SIM of both PML and HIPK2 is necessary for the recruitment of HIPK2 to PML NBs [43], [51]. Functionally, the SIM-dependent targeting of HIPK2 to PML NBs is crucial for HIPK2-mediated p53 activation and induction of apoptosis [51]. Interestingly, MageA2, a melanoma antigen gene product, whose expression decreases cellular senescence and increases proliferation, binds to all nuclear PML except to PMLVI [52] raising the question of the potential involvement of the SIM in these interactions. Also, the SIM of PMLI plays a key role in antiviral defence as an intact SIM is required for restriction of HSV-1 infection [53], [54]. During HSV-1 infection, one of the first viral protein to be expressed is ICP0 (infected cell protein 0). Like RNF4, ICP0 has properties related to those of cellular STUbLs as it induces the proteasome-dependent degradation of SUMO-conjugated proteins such as PML during HSV-1 infection [55]. Whether the SIM of PML contributes to the ability of ICP0 to counteract host-cell intrinsic resistance to HSV-1 infection is presently unknown. Here, we suggest that the SIM of PML, that is required for PML degradation in response to As_2_O_3_, could promote the direct recruitment of SUMO-modified intrinsic proteins of the proteasome complex or bind SUMO-modified proteins that indirectly target the proteasome to PML NBs.

Taken together our results show that PML SUMOylation and interaction with RNF4, which were both enhanced in response to As_2_O_3_, were not sufficient to induce the degradation of all PML isoforms. In addition to the poly-SUMOylation of PML that is needed to recruit RNF4, the SIM of PML is required to favor PML ubiquitination and the recruitment of the 20S core and the β regulatory subunit of 11S of the proteasome within PML NBs. These events lead to As_2_O_3_-induced PML proteasomal degradation. It is not yet clear why resistance to degradation of PMLVI due to the absence of the SIM was partially overcome in As_2_O_3_-treated cells only when RNF4 was overexpressed. We suggest that RNF4 is limiting in cells and that its exogenous expression could increase its interaction with PML thus facilitating the recruitment of the ubiquitin machinery.

Altogether, this study shows that the differential regulation of the degradation of nuclear PML isoforms is dependent on the SIM present on PMLI to PMLV but not on PMLVI. Thus, both the covalent conjugation of SUMO to PML [4], [5], [10], [16] and the non-covalent interactions with PML SIM are required for RNF4- or As_2_O_3_-induced proteasome-dependent PML degradation.

## Materials and Methods

### IFN, As_2_O_3_ and Antibodies

Human recombinant IFNγ was from Roussel Uclaf. A stock solution of As_2_O_3_ (Sigma) was prepared in 1 M NaOH, diluted in growth medium and used at the concentration and the time indicated in the figure legends. Anti-Alexa-Fluor 488-conjugated secondary antibody was from Invitrogen. Rabbit polyclonal (H-238) and mouse (PGM3) anti-PML antibodies were from Santa-Cruz Biotechnology, monoclonal anti-luciferase antibody was from ABCys, rabbit polyclonal antibodies against proteasome 20S core and against β regulatory subunit of 11S (PA28) were from Biomol international, mouse anti-ubiquitin antibody were from Novus Biologicals, HRP-conjugate monoclonal anti-actin and anti-FLAG (clone M2) antibodies were from Sigma, and anti-polyclonal histone H3 antibody was from Cell Signaling.

### Constructs and Expression Vectors

#### PML and RNF4 constructs

cDNA of all PML isoforms (I, II, III, IV, V, VI and VII) were amplified by PCR from pBluescript SK^+^ for subcloning in appropriate vectors. The accession numbers (GenBank) for PML isoforms are AF230401 (PMLI), AF230403 (PMLII), S50913 (PMLIII), AF230406 (PMLIV), AF230402 (PMLV), AF230405 (PMLVI), AF230408 (PMLVII). To generate untagged expression constructs, PML PCR fragments of each isoform were digested respectively by BamHI and XhoI for PMLIII, IV, V, VI, and VII, by BamHI and Xba1 for PMLI and by HindIII and XhoI for PMLII (“PML_BamHI_ATG sense” primer was used as sense primer for all isoforms; specific antisense primers were designed for each isoform that were called PMLX _Stop_Enz name where “X” is the number of the isoform and “Enz name” is the name of the enzyme used to cut cDNA in 3′). PMLIII-SIM mutant (previously named PML_SBD_ and mutated at the hydrophobic core sequence VVVI) (amino acids 556 to 559) is as described (16). pFLAG-RNF4 was a kind gift from F.J. Kaiser and B. Horsthemke [56].

### BRET Constructs

YFP-SUMO1/P63165, YFP-SUMO2/P61956 and YFP-SUMO3/P55854 (all cloned in pEYFP(C1), BD Biosciences Clontech) were kind gifts of Dr M. Dasso (NIH, Bethesda, USA) [57]. For PML and RNF4 BRET constructs, Luc or YFP were cloned together with the cDNA of interest by a three-piece-ligation in the BamHI/XhoI or BamH1/XbaI site of pcDNA3.1 (+) (Invitrogen). Luc was amplified by PCR from pcDNA3-CXCR4-Luc vector [16] and cloned at the N-terminal of the cDNA of interest as a BamH1-EcoR1 fragment (primers used: “Luc_BamHI_ATG_N-term sense” and “Luc_noSTOP_EcoR1_N-term antisense”). YFP was amplified by PCR from pcDNA3-CXCR4-YFP [16], digested with EcoRI and XhoI, and cloned at the C-terminal of the cDNA of interest (primers used: “YFP_EcoRI_ATG_C-term sense” and “YFP_STOP_XhoI_C-term antisense”). To generate Luc-PML isoform BRET constructs, the cDNA of interest was amplified by PCR for cloning in phase at the C terminal of Luc as an EcoRI-XhoI (PMLVII) or EcoRI-XbaI (PMLIII, PMLIV, PMLV, PMLVI), AgeI/SalI (PMLI) or AgeI/XhoI (PMLII) fragment (primers used: “PML_BamHI_ATG sense” and “Fusion-PMLX Enz name 1/Enz name 2_antisense” where “X” is the number of the isoform and “Enz name 1″ is the name of the enzyme used to cut cDNA and Enz name 2 is the name of the compatible cloning site in the vector). To generate RNF4-YFP BRET construct, RNF4 was PCR amplified from pFLAG-RNF4 and cloned in phase, as an BamHI-EcoRI fragment, N-terminal to YFP in the BamHI-XhoI site of pcDNA3.1 owing to a tree-piece-ligation (primers used: “RNF4_BamHI_N-term sense” and a “RNF4-fusion_EcoRI_ antisense”). In each case, the sequence joining the cDNA of interest and either Luc or YFP sequence encodes VPVNSGGGGS as a linker.

To compare the interaction of RNF4 with the different PML isoforms, which differ in their C-terminal region, each PML isoform tagged at its N-terminal region with luciferase in pair with RNF4-YFP.

### Primers

#### PML primers

*Sense primers-*

PML_BamHI_ATG sense :

5′TGTCTAAGCTTGCTAGCGGATCCACACCATGGAGCCTGCACCCGCCCGATCTCCG3′;

Fusion-PML sense (AgeI or EcoRI):

5′CTGCTACCGGTGAATTCTGGTGGAGGCATGGAGCCTGCAGCCCGATCTCC3′;

*Antisense primers-*

PMLI Stop XbaI

5′AATACGGTACCTCTAGAACTCAGCTCTGCTGGGAGGCCCTCTC3′;

PMLII_Stop_XhoI

5′AATACCTCGAGTCTAGATATCAGAGGCCTGCTTGACGGGCGCCTGGGAC3′;

PMLIII_Stop_XhoI

5′ATCCGCCTCGAGCAGAATCAGCGGGCTGGTGGGGAGGCCAAGC3′;

PMLIV_Stop_XhoI

5′AATACCTCGAGTCTAGATACTAAATTAGAAAGGGGTGGGGGTAGCCCCAGG3′,

PMLV_Stop_XhoI

5′AATACCTCGAGTCTAGATATCAATGCCTCACTGGAAAATTCCCCAGGCGC3′,

PMLVI_Stop_XhoI

5′AATACCTCGAGTCTAGATATCACCACAACGCGTTCCTCTCCCTACCTGCC3′;

PMLVII Stop_XhoI

5′GTACCTCGAGTATTAATGAGTGCTACTCTGTGCAGGGCCTGTAAGAGCATGGGCTGGAGGAGGCACCAGGTCAACGTCAATAGGGTCCCTGGGAGTGC 3′;

Fusion-PMLI AgeI/SalI_antisense

5′AATACGGTACCGTCGACACTCAGCTCTGCTGGGAGGCCCTCTC3′;

Fusion-PMLII AgeI/XhoI_antisense

5′AATACCTCGAGTCTAGATATCAGAGGCCTGCTTGACGGGCGCCTGGGAC3′;

Fusion-PMLIII EcoRI/XbaI_antisense

5′ATCTAGTCTAGACCGCTCGAGCGGTCAGCGGGCTGGTGGGGAGGCC3′;

Fusion-PMLIV EcoRI/XbaI_antisense

5′AATACCTCGAGTCTAGATACTAAATTAGAAAGGGGTGGGGGTAGCCCCAGG3′;

Fusion-PMLV EcoRI/XbaI_antisense

5′AATACCTCGAGTCTAGATATCAATGCCTCACTGGAAAATTCCCCAGGCGC3′;

Fusion-PMLVI EcoRI/XbaI_antisense

5′AATACCTCGAGTCTAGATATCACCACAACGCGTTCCTCTCCCTACCTGCC3′;

Fusion-PMLVII EcoRI/XhoI_antisense

5′GTACCTCGAGTATTAATGAGTGCTACTCTGTGCAGGGCCTGTAAGAGCATGGGCTGGAGGAGGCACCAGGTCAACGTCAATAGGGTCCCTGGGAGTGC3′;

*RNF4 primers:*

RNF4_BamHI_N-term sense

5′TGTCTAAGCTTGCTAGCGGATCCACACCATGAGTACAAGAAAGCGTCGTGGTGG3′;

RNF4-fusion_EcoRI_antisense 5′CACCAGAATTCACCGGTACTATATAAATGGGATGGTACCGTTTATGG3′;

*Luc and YFP primers-*

Luc_BamHI_ATG_N-term sense:

5′CTGTGCTAGCGGATCCATAATGACCAGCAAGGTGTACGACCCCGAGC-3′;

Luc_noSTOP_EcoR1_N-term antisense:

5′TCCACCAGAATTCACCGGTACCTGCTCGTTCTTCAGCACTCTCTCCACG3′;

YFP_EcoRI_ATG_C-term sense:

5′ACCGGTGAATTCTGGTGGAGGCGGATCTATGGTGAGCAAGGGCGAGGAGCTG3′;

YFP_STOP_XhoI_C-term antisense:

5′CTCTAGACTCGAGCGGCCGCTTTACTTGTACAGCTCGTCCATGCCG3′;

### Cell Cultures

Human glioblastoma astrocytoma U373MG**,** human embryonic kidney HEK293T cells (American Type Culture Collection, Rockville, MD) as well as mouse embryonic fibroblasts (MEFs) from knockout PML (PML^−/−^) mice [58] immortalized by the simian virus 40 (SV40) T antigen were grown at 37°C in DMEM supplemented with 10% foetal calf serum. U373MG cells transfected with empty vector or stably expressing individual PML isoforms (PMLI to VII) were kept in medium supplemented with 0.5 mg/ml of neomycin.

### Stable Expression of PML Isoforms

Stable U373MG cells expressing each of the PML isoforms (PMLI to VII) or PMLIII-SIM were obtained via transfection with constructs corresponding to each of these isoforms cloned in pcDNA3.1 and subsequent neomycin selection at a final concentration of 0.5 mg/ml. Controls cells were generated in the same way using the empty vectors.

### BRET Transient Transfections

HEK293T cells were seeded at a density of 5×10^5^ cells per well in 6-well dishes, 24 h before transfection. Transient transfections were performed using Polyethylenimine (PEI) (Polysciences, Inc., Warrington, PA) in Optimem medium. PEI powder was dissolved to a concentration of 1 mg/ml in water, which had been heated to 80°C. Usually, 0.1 µg of Luc-PML construct was transfected alone or with increasing quantities of YFP-tagged SUMO1. The amount of transfected DNA was completed to a total of 2 µg with pcDNA3.1 (+) empty vector. PEI (10 µg in 100 µl of Optimem medium) was added on the DNA and the samples were incubated for 20 min at room temperature. The PEI-DNA suspension was then added to the attached cells in 2 ml of fresh culture media. Following an overnight incubation, the transfection medium was replaced with complete DMEM for 3 h to allow cell recovery. Transfected cells (8×10^4^) were then detached and replated in 96-wells white plates with clear bottom (Costar) pre-treated with D-polylysine (Sigma) and left in culture for 24 h before being processed for BRET assay. When required, the plated cells were treated with As_2_O_3_ at 37°C at the indicated times in figure legends.

### BRET Detection

BRET measurements were done on attached cells as previously described [16]. Just before measurement, culture media was replaced by PBS and coelenterazine H (Nanolight Technology) was added to a final concentration of 5 µM. Readings were then collected using a multi-mode microplate reader Mithras LB940 (Berthold) allowing the sequential integration of the signals detected in the 480±20 nm and 530±20 nm windows, for luciferase and YFP light emissions, respectively. The BRET signal is determined by calculating the ratio of the light intensity emitted by the YFP fusion (acceptor) over the light intensity emitted by the Luc fusion (donor). The values were corrected by subtracting the background BRET signal detected when the Luc fusion construct was expressed alone. For BRET titration experiments, BRET ratios were expressed as a function of the [acceptor]/[donor] expression ratio (YFP/Luc). Expression level of the acceptor and donor was determined by direct measurement of total fluorescence of the YFP fusion and luminescence of Luc fusion. Total fluorescence was determined with Mithras LB940 using an excitation filter at 485 nM and an emission filter at 535 nM. Total luminescence was measured in the Mithras LB940, 10 min after the addition of coelenterazine H and the reading was taken in the absence of emission filter.

### Immunofluorescence Analysis

Cells grown on glass coverslip were fixed with 4% paraformaldehyde and permeabilized in 0.1% TRITON X-100 for anti-PML and with acetone for anti-proteasome 20S core and anti-β regulatory subunit of 11S. Cells were then prepared for immunofluorescence staining and analyzed by confocal microscopy. PML was detected with rabbit or mouse anti-PML antibodies and the corresponding anti-IgG antibody conjugated to Alexa 488 or 594. The rabbit polyclonal antibodies against proteasome 20S core and against β regulatory subunit of 11S (PA28) (Biomol international) were used for detection of proteasome components followed by Alexa 488. The cells were mounted onto glass slides by using Immu-Mount (Shandon) containing 4,6-diamidino-2-phenylindole (DAPI) to stain nuclei. Confocal laser microscopy was performed on a Leica SP2 microscope.

### Immunoprecipitation Assays

Transfected cells (10^7^) were incubated for 30 min at 4°C in 0.5 ml of buffer containing 20 mM Tris-HCl pH 7.4, 1 M NaCl, 5 mM MgCl_2_, 1% triton, and 1 mM phenylmethylsulfonyl fluoride (PMSF). An aliquot was saved for the input analysis by Western blot. After cell lysis, 1.25 ml of immuno-precipitation buffer (IB) (20 mM Tris-HCl pH 7.4, 150 mM NaCl, 0.5% DOC, 1% Triton X-100, 0.1% SDS and 1 mM EDTA) was added. Immunoprecipitation of PML was carried out by incubation the samples overnight at 4°C in the presence of rabbit anti-PML antibody. Protein G beads (Sigma) were then added and the samples were mixed for 2 h at room temperature. The beads were collected, washed four times with modified IB buffer (5 mM Tris-HCl pH 7.4) and the bound proteins were subjected to Western blot analysis. Immunoprecipitation of Flag-tagged proteins was carried out using anti-FLAG M2 affinity gel (Sigma) as recommended by the manufacturer. Briefly, 40 µl of the resin were added to each sample and incubated overnight at 4°C. The resin was washed three times with TBS and the bound proteins eluted in Laemmli buffer were subjected to Western blot analysis.

### Western Blot Analysis

For total cell extracts, cells were washed and re-suspended in PBS, lysed in hot Laemmli sample buffer and boiled for 5 min. Cytoplasmic (Cyt) and nuclear (Nu) extracts were prepared as previously described [59]. About 20 µg of protein was analyzed on a 10% SDS-PAGE gel, and transferred onto a nitrocellulose membrane. The membranes were treated with 5% skimmed milk in TBS for 2 h and incubated overnight at 4°C with rabbit polyclonal anti-PML (clone H-238) or anti-actin antibodies. The blots were then washed extensively in PBS-Tween and incubated for 1 h with the appropriate peroxidase-coupled secondary antibodies (Amersham). All of the blots were revealed by chemoluminescence (ECL, Amersham).

## Supporting Information

Figure S1 **BRET dose-response curves to As_2_O_3_ treatment for detecting the increased SUMOylation by SUMO1, SUMO2 or SUMO3 of nuclear PML isoforms.** HEK293T cells, transiently transfected with a fixed amount of a Luc-PML fusion (PMLI to VII) and a fixed amount of YFP-SUMO1, YFP-SUMO2 or YFP-SUMO3, were treated at different doses of As_2_O_3_ during 4 h and used for BRET assays. As previously demonstrated [40], the close proximity of several YFP moieties could cause quenching or interference phenomena that could lead to a decrease in the BRET signal. This explains that a stronger interaction is not observed with YFP-SUMO2 or -SUMO3 than with YFP-SUMO1 in response to As_2_O_3_.
(TIF)

Figure S2 **Detection by BRET of the increase in the SUMOylation of nuclear PML isoforms by SUMO2 or SUMO3 in response to As_2_O_3_ in living cells.** HEK293T cells, transiently transfected with a fixed amount of a Luc-PML fusion (PML I to VII) and increasing amounts of YFP-SUMO2 (A) or YFP-SUMO3 (B), were treated in the presence or absence of As_2_O_3_ (5 µM, 4 h) and used for BRET titration assays. BRET saturation curves are presented for each PML isoform in the absence (open square) or presence of As_2_O_3_ (open square).
(TIF)

Figure S3 **Immunofluorescence and Western blot analysis of PML isoforms.** (A, B) Localization and expression of Luc-PML isoforms**.** HEK293T cells, transiently transfected with the empty vector or a Luc-PML fusion (PMLIII,V,VI or VII) were analyzed by confocal microscopy using anti-PML antibody (A) or by Western blot using anti-Luc or anti-actin antibodies (B). (C) The SIM of PML was not required for As_2_O_3_-induced PML SUMOylation. U373MG cells stably expressing PMLIII-SIM were treated with of 5 μM of As_2_O_3_ for 4 h. Total cell extracts were analyzed by Western blot for PML and actin expression. The unmodified PML isoforms are indicated by arrowheads and the modified PML species by brackets. (D) Analysis of cytoplasmic and nuclear extracts. U373MG cells prepared in duplicate were treated with 1000 units/ml of IFNγ. One day later As_2_O_3_ was added in one sample to a final concentration of 5 µM for 24 h. Cytoplasmic (Cyt) and nuclear (Nu) extracts were analyzed by Western blot with anti-PML, anti-actin and anti-histone H3 antibodies.
(TIF)

Figure S4 **Interaction by BRET of RNF4 with SUMOylated PML isoforms (I, II, IV, V or VII).** (A–C) HEK293T cells, transiently transfected with a Luc-PML fusion (I, II, IV, V or VII) and increasing amounts of RNF4-YFP, were treated in the presence or absence of with As_2_O_3_ (5 µM, 4 h) and used for BRET titration assays. BRET saturation curves in the absence (open square) or presence of As_2_O_3_ (closed square) are presented for each PML isoform in A) for PMLI and PMLII, B) for PMLIV and PMLV and C) for PMLVII. Bar graphs are for presenting the BRET signal (BRET), the luciferase expression and YFP expression of individual untreated (gray bars) and treated (black bars) samples; in the presence of RNF4-YFP, the expression of the Luc-PML fusion was decreased by the As_2_O_3_ treatment whereas the expression of RNF4-YFP stayed relatively constant.
(TIF)

Figure S5 **Requirement of the SIM for the recruitment of the 20S proteasome to PML NBs in response to As_2_O_3_.** Confocal immunofluorescence analysis of PML and 20S core proteasome were performed on U373MG cells, stably expressing PMLIII, PMLIII-SIM or PMLVI, treated or not for 1 h with 5 μM of As_2_O_3_. PML and 20S core were detected with a mouse anti-PML and a rabbit anti-20S antibodies followed by the corresponding anti-IgG antibody conjugated to Alexa 594 (red) and 488 (green), respectively. As reported previously [5] colocalization between PMLIII and the 20S core is observed in some cells only in response to As_2_O_3_. The merged images revealed co-localization of PML and endogenous 20S core proteasome only for PMLIII in the presence of As_2_O_3_.
(TIF)

## References

[1] Ishov AM, Sotnikov AG, Negorev D, Vladimirova OV, Neff N, et al (1999) PML is critical for ND10 formation and recruits the PML-interacting protein daxx to this nuclear structure when modified by SUMO-1. J Cell Biol 147: 221–234. 10525530 10.1083/jcb.147.2.221 PMC2174231

[2] Geoffroy MC, Chelbi-Alix MK (2011) Role of promyelocytic leukemia protein in host antiviral defense. J Interferon Cytokine Res 31: 145–158. 21198351 10.1089/jir.2010.0111

[3] Bernardi R, Papa A, Pandolfi PP (2008) Regulation of apoptosis by PML and the PML-NBs. Oncogene 27: 6299–6312. 18931695 10.1038/onc.2008.305

[4] Tatham MH, Geoffroy MC, Shen L, Plechanovova A, Hattersley N, et al (2008) RNF4 is a poly-SUMO-specific E3 ubiquitin ligase required for arsenic-induced PML degradation. Nat Cell Biol 10: 538–546. 18408734 10.1038/ncb1716

[5] Lallemand-Breitenbach V, Zhu J, Puvion F, Koken M, Honore N, et al (2001) Role of promyelocytic leukemia (PML) sumolation in nuclear body formation, 11S proteasome recruitment, and As2O3-induced PML or PML/retinoic acid receptor alpha degradation. J Exp Med 193: 1361–1371. 11413191 10.1084/jem.193.12.1361 PMC2193303

[6] Pampin M, Simonin Y, Blondel B, Percherancier Y, Chelbi-Alix MK (2006) Cross talk between PML and p53 during poliovirus infection: implications for antiviral defense. J Virol 80: 8582–8592. 16912307 10.1128/JVI.00031-06 PMC1563870

[7] El McHichi B, Regad T, Maroui MA, Rodriguez MS, Aminev A, et al (2010) SUMOylation promotes PML degradation during encephalomyocarditis virus infection. J Virol 84: 11634–11645. 20826694 10.1128/JVI.01321-10 PMC2977872

[8] Everett RD, Chelbi-Alix MK (2007) PML and PML nuclear bodies: implications in antiviral defence. Biochimie 89: 819–830. 17343971 10.1016/j.biochi.2007.01.004

[9] Krieghoff-Henning E, Hofmann TG (2008) Role of nuclear bodies in apoptosis signalling. Biochim Biophys Acta 1783: 2185–2194. 18680765 10.1016/j.bbamcr.2008.07.002

[10] Lallemand-Breitenbach V, Jeanne M, Benhenda S, Nasr R, Lei M, et al (2008) Arsenic degrades PML or PML-RARalpha through a SUMO-triggered RNF4/ubiquitin-mediated pathway. Nat Cell Biol 10: 547–555. 18408733 10.1038/ncb1717

[11] Van Damme E, Laukens K, Dang TH, Van Ostade X (2010) A manually curated network of the PML nuclear body interactome reveals an important role for PML-NBs in SUMOylation dynamics. Int J Biol Sci 6: 51–67. 20087442 10.7150/ijbs.6.51 PMC2808052

[12] Sachdev S, Bruhn L, Sieber H, Pichler A, Melchior F, et al (2001) PIASy, a nuclear matrix-associated SUMO E3 ligase, represses LEF1 activity by sequestration into nuclear bodies. Genes Dev 15: 3088–3103. 11731474 10.1101/gad.944801 PMC312834

[13] Best JL, Ganiatsas S, Agarwal S, Changou A, Salomoni P, et al (2002) SUMO-1 protease-1 regulates gene transcription through PML. Mol Cell 10: 843–855. 12419228 10.1016/s1097-2765(02)00699-8

[14] Kamitani T, Kito K, Nguyen HP, Wada H, Fukuda-Kamitani T, et al (1998) Identification of three major sentrinization sites in PML. J Biol Chem 273: 26675–26682. 9756909 10.1074/jbc.273.41.26675

[15] Muller S, Matunis MJ, Dejean A (1998) Conjugation with the ubiquitin-related modifier SUMO-1 regulates the partitioning of PML within the nucleus. EMBO J 17: 61–70. 9427741 10.1093/emboj/17.1.61 PMC1170358

[16] Percherancier Y, Germain-Desprez D, Galisson F, Mascle XH, Dianoux L, et al (2009) Role of SUMO in RNF4-mediated promyelocytic leukemia protein (PML) degradation: sumoylation of PML and phospho-switch control of its SUMO binding domain dissected in living cells. J Biol Chem 284: 16595–16608. 19380586 10.1074/jbc.M109.006387 PMC2713554

[17] Zhu J, Koken MH, Quignon F, Chelbi-Alix MK, Degos L, et al (1997) Arsenic-induced PML targeting onto nuclear bodies: implications for the treatment of acute promyelocytic leukemia. Proc Natl Acad Sci U S A 94: 3978–3983. 9108090 10.1073/pnas.94.8.3978 PMC20553

[18] Hayakawa F, Privalsky ML (2004) Phosphorylation of PML by mitogen-activated protein kinases plays a key role in arsenic trioxide-mediated apoptosis. Cancer Cell 5: 389–401. 15093545 10.1016/s1535-6108(04)00082-0

[19] Zhang XW, Yan XJ, Zhou ZR, Yang FF, Wu ZY, et al (2010) Arsenic trioxide controls the fate of the PML-RARalpha oncoprotein by directly binding PML. Science 328: 240–243. 20378816 10.1126/science.1183424

[20] Weisshaar SR, Keusekotten K, Krause A, Horst C, Springer HM, et al (2008) Arsenic trioxide stimulates SUMO-2/3 modification leading to RNF4-dependent proteolytic targeting of PML. FEBS Lett 582: 3174–3178. 18708055 10.1016/j.febslet.2008.08.008

[21] Perry JJ, Tainer JA, Boddy MN (2008) A SIM-ultaneous role for SUMO and ubiquitin. Trends Biochem Sci 33: 201–208. 18403209 10.1016/j.tibs.2008.02.001

[22] Chiariotti L, Benvenuto G, Fedele M, Santoro M, Simeone A, et al (1998) Identification and characterization of a novel RING-finger gene (RNF4) mapping at 4p16.3. Genomics 47: 258–265. 9479498 10.1006/geno.1997.5105

[23] Hakli M, Karvonen U, Janne OA, Palvimo JJ (2005) SUMO-1 promotes association of SNURF (RNF4) with PML nuclear bodies. Exp Cell Res 304: 224–233. 15707587 10.1016/j.yexcr.2004.10.029

[24] Moilanen AM, Poukka H, Karvonen U, Hakli M, Janne OA, et al (1998) Identification of a novel RING finger protein as a coregulator in steroid receptor-mediated gene transcription. Mol Cell Biol 18: 5128–5139. 9710597 10.1128/mcb.18.9.5128 PMC109098

[25] Rabellino A, Carter BJ, Konstantinidou G, Shwu-Yuan W, Rimessi A, et al.. (2012) The SUMO E3-ligase PIAS1 regulates the tumor suppressor PML and its oncogenic counterpart PML-RARA. Cancer Res [Epub ahead of print]. 10.1158/0008-5472.CAN-11-3159 PMC3342450 22406621

[26] Jensen K, Shiels C, Freemont PS (2001) PML protein isoforms and the RBCC/TRIM motif. Oncogene 20: 7223–7233. 11704850 10.1038/sj.onc.1204765

[27] Bernardi R, Pandolfi PP (2007) Structure, dynamics and functions of promyelocytic leukaemia nuclear bodies. Nat Rev Mol Cell Biol 8: 1006–1016. 17928811 10.1038/nrm2277

[28] Wei X, Yu ZK, Ramalingam A, Grossman SR, Yu JH, et al (2003) Physical and functional interactions between PML and MDM2. J Biol Chem 278: 29288–29297. 12759344 10.1074/jbc.M212215200

[29] Fogal V, Gostissa M, Sandy P, Zacchi P, Sternsdorf T, et al (2000) Regulation of p53 activity in nuclear bodies by a specific PML isoform. EMBO J 19: 6185–6195. 11080164 10.1093/emboj/19.22.6185 PMC305840

[30] Henderson BR, Eleftheriou A (2000) A comparison of the activity, sequence specificity, and CRM1-dependence of different nuclear export signals. Exp Cell Res 256: 213–224. 10739668 10.1006/excr.2000.4825

[31] Condemine W, Takahashi Y, Le Bras M, de The H (2007) A nucleolar targeting signal in PML-I addresses PML to nucleolar caps in stressed or senescent cells. J Cell Sci 120: 3219–3227. 17878236 10.1242/jcs.007492

[32] Shen TH, Lin HK, Scaglioni PP, Yung TM, Pandolfi PP (2006) The mechanisms of PML-nuclear body formation. Mol Cell 24: 331–339. 17081985 10.1016/j.molcel.2006.09.013 PMC1978182

[33] Stehmeier P, Muller S (2009) Phospho-regulated SUMO interaction modules connect the SUMO system to CK2 signaling. Mol Cell 33: 400–409. 19217413 10.1016/j.molcel.2009.01.013

[34] Condemine W, Takahashi Y, Zhu J, Puvion-Dutilleul F, Guegan S, et al (2006) Characterization of endogenous human promyelocytic leukemia isoforms. Cancer Res 66: 6192–6198. 16778193 10.1158/0008-5472.CAN-05-3792

[35] Brand P, Lenser T, Hemmerich P (2010) Assembly dynamics of PML nuclear bodies in living cells. PMC Biophys 3: 3. 20205709 10.1186/1757-5036-3-3 PMC2854101

[36] Germain-Desprez D, Bazinet M, Bouvier M, Aubry M (2003) Oligomerization of transcriptional intermediary factor 1 regulators and interaction with ZNF74 nuclear matrix protein revealed by bioluminescence resonance energy transfer in living cells. J Biol Chem 278: 22367–22373. 12684500 10.1074/jbc.M302234200

[37] Xu Y, Piston DW, Johnson CH (1999) A bioluminescence resonance energy transfer (BRET) system: application to interacting circadian clock proteins. Proc Natl Acad Sci U S A 96: 151–156. 9874787 10.1073/pnas.96.1.151 PMC15108

[38] Angers S, Salahpour A, Joly E, Hilairet S, Chelsky D, et al (2000) Detection of beta 2-adrenergic receptor dimerization in living cells using bioluminescence resonance energy transfer (BRET). Proc Natl Acad Sci U S A 97: 3684–3689. 10725388 10.1073/pnas.060590697 PMC16300

[39] Blondel D, Kheddache S, Lahaye X, Dianoux L, Chelbi-Alix MK (2010) Resistance to rabies virus infection conferred by the PMLIV isoform. J Virol 84: 10719–10726. 20702643 10.1128/JVI.01286-10 PMC2950589

[40] Perroy J, Pontier S, Charest PG, Aubry M, Bouvier M (2004) Real-time monitoring of ubiquitination in living cells by BRET. Nat Methods 1: 203–208. 15782195 10.1038/nmeth722

[41] Nervi C, Ferrara FF, Fanelli M, Rippo MR, Tomassini B, et al (1998) Caspases mediate retinoic acid-induced degradation of the acute promyelocytic leukemia PML/RARalpha fusion protein. Blood 92: 2244–2251. 9746761

[42] Chelbi-Alix MK, Pelicano L, Quignon F, Koken MH, Venturini L, et al (1995) Induction of the PML protein by interferons in normal and APL cells. Leukemia 9: 2027–2033. 8609713

[43] de la Vega L, Frobius K, Moreno R, Calzado MA, Geng H, et al (2011) Control of nuclear HIPK2 localization and function by a SUMO interaction motif. Biochim Biophys Acta 1813: 283–297. 21145359 10.1016/j.bbamcr.2010.11.022

[44] Lin DY, Huang YS, Jeng JC, Kuo HY, Chang CC, et al (2006) Role of SUMO-interacting motif in Daxx SUMO modification, subnuclear localization, and repression of sumoylated transcription factors. Mol Cell 24: 341–354. 17081986 10.1016/j.molcel.2006.10.019

[45] Zhu J, Zhu S, Guzzo CM, Ellis NA, Sung KS, et al (2008) Small ubiquitin-related modifier (SUMO) binding determines substrate recognition and paralog-selective SUMO modification. J Biol Chem 283: 29405–29415. 18708356 10.1074/jbc.M803632200 PMC2570875

[46] Chang CC, Naik MT, Huang YS, Jeng JC, Liao PH, et al (2011) Structural and Functional Roles of Daxx SIM Phosphorylation in SUMO Paralog-Selective Binding and Apoptosis Modulation. Mol Cell 42: 62–74. 21474068 10.1016/j.molcel.2011.02.022

[47] Escobar-Cabrera E, Okon M, Lau DK, Dart CF, Bonvin AM, et al (2011) Characterizing the N- and C-terminal Small ubiquitin-like modifier (SUMO)-interacting motifs of the scaffold protein DAXX. J Biol Chem 286: 19816–19829. 21383010 10.1074/jbc.M111.231647 PMC3103359

[48] Geoffroy MC, Jaffray EG, Walker KJ, Hay RT (2010) Arsenic-induced SUMO-dependent recruitment of RNF4 into PML nuclear bodies. Mol Biol Cell 21: 4227–4239. 20943951 10.1091/mbc.E10-05-0449 PMC2993750

[49] Dacres H, Dumancic MM, Horne I, Trowell SC (2009) Direct comparison of fluorescence- and bioluminescence-based resonance energy transfer methods for real-time monitoring of thrombin-catalysed proteolytic cleavage. Biosens Bioelectron 24: 1164–1170. 18723336 10.1016/j.bios.2008.07.021

[50] Dacres H, Dumancic MM, Horne I, Trowell SC (2009) Direct comparison of bioluminescence-based resonance energy transfer methods for monitoring of proteolytic cleavage. Anal Biochem 385: 194–202. 19026607 10.1016/j.ab.2008.10.040

[51] Sung KS, Lee YA, Kim ET, Lee SR, Ahn JH, et al (2011) Role of the SUMO-interacting motif in HIPK2 targeting to the PML nuclear bodies and regulation of p53. Exp Cell Res 317: 1060–1070. 21192925 10.1016/j.yexcr.2010.12.016

[52] Peche LY, Scolz M, Ladelfa MF, Monte M, Schneider C (2011) MageA2 restrains cellular senescence by targeting the function of PMLIV/p53 axis at the PML-NBs. Cell Death Differ. 10.1038/cdd.2011.173 PMC3354046 22117195

[53] Cuchet D, Sykes A, Nicolas A, Orr A, Murray J, et al (2011) PML isoforms I and II participate in PML-dependent restriction of HSV-1 replication. J Cell Sci 124: 280–291. 21172801 10.1242/jcs.075390 PMC3010193

[54] Cuchet-Lourenco D, Boutell C, Lukashchuk V, Grant K, Sykes A, et al (2011) SUMO pathway dependent recruitment of cellular repressors to herpes simplex virus type 1 genomes. PLoS Pathog 7: e1002123. 21779164 10.1371/journal.ppat.1002123 PMC3136452

[55] Boutell C, Cuchet-Lourenco D, Vanni E, Orr A, Glass M, et al (2011) A viral ubiquitin ligase has substrate preferential SUMO targeted ubiquitin ligase activity that counteracts intrinsic antiviral defence. PLoS Pathog 7: e1002245. 21949651 10.1371/journal.ppat.1002245 PMC3174244

[56] Kaiser FJ, Moroy T, Chang GT, Horsthemke B, Ludecke HJ (2003) The RING finger protein RNF4, a co-regulator of transcription, interacts with the TRPS1 transcription factor. J Biol Chem 278: 38780–38785. 12885770 10.1074/jbc.M306259200

[57] Ayaydin F, Dasso M (2004) Distinct in vivo dynamics of vertebrate SUMO paralogues. Mol Biol Cell 15: 5208–5218. 15456902 10.1091/mbc.E04-07-0589 PMC532004

[58] Wang ZG, Ruggero D, Ronchetti S, Zhong S, Gaboli M, et al (1998) PML is essential for multiple apoptotic pathways. Nat Genet 20: 266–272. 9806545 10.1038/3073

[59] Galisson F, Mahrouche L, Courcelles M, Bonneil E, Meloche S, et al.. (2010) A novel proteomics approach to identify SUMOylated proteins and their modification sites in human cells. Mol Cell Proteomics. 10.1074/mcp.M110.004796 PMC3033685 21098080

