# Supplementary figures and images for: Requirement of PML SUMO Interacting Motif for RNF4- or Arsenic Trioxide-Induced Degradation of Nuclear PML Isoforms
Source: PLoS One. 2012 Sep 18;7(9):e44949. doi: 10.1371/journal.pone.0044949 (PMC3445614; doi:10.1371/journal.pone.0044949)

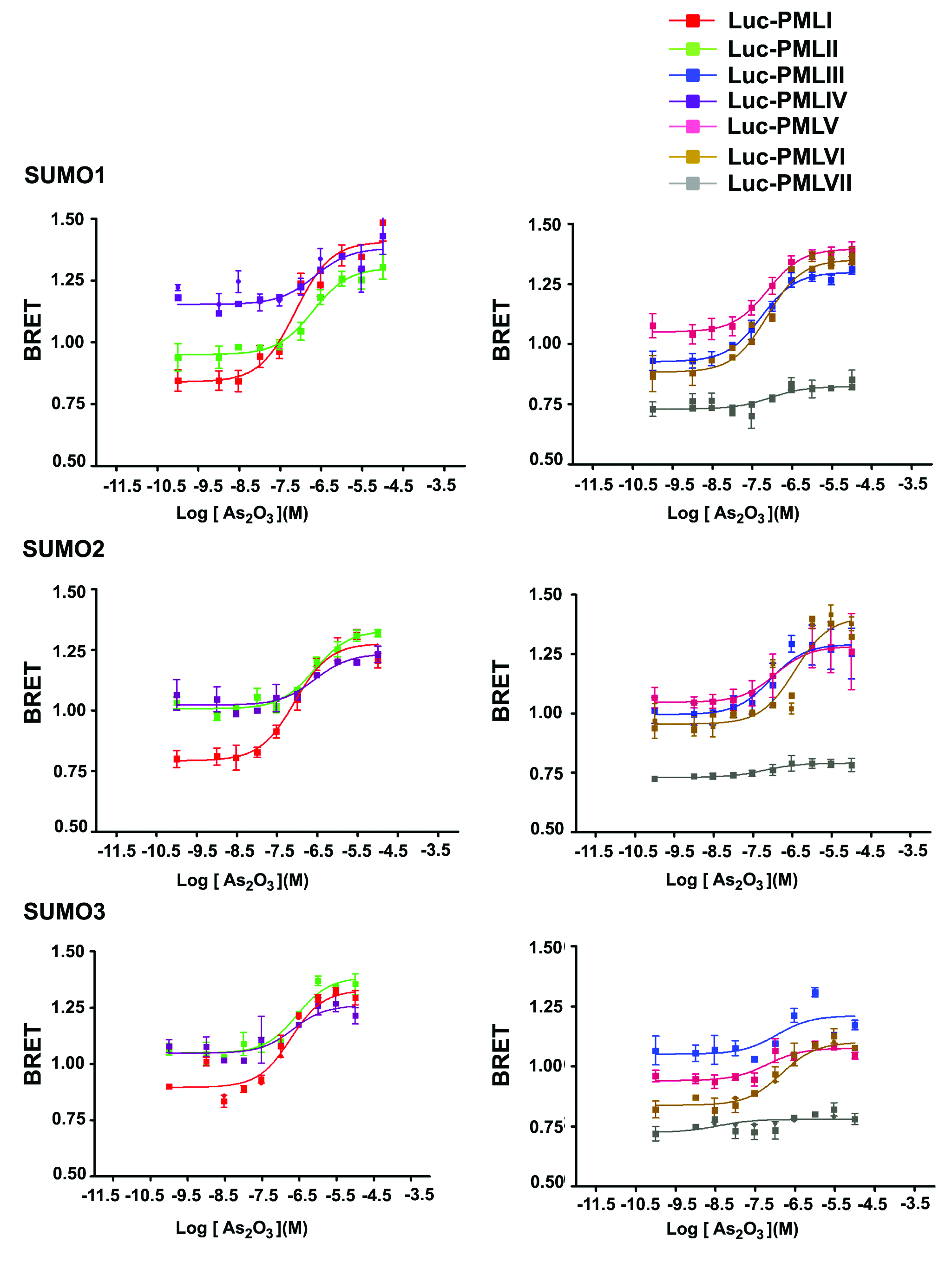

Supplement: Figure S1 — BRET dose-response curves to As2O3 treatment for detecting the increased SUMOylation by SUMO1, SUMO2 or SUMO3 of nuclear PML isoforms. HEK293T cells, transiently transfected with a fixed amount of a Luc-PML fusion (PMLI to VII) and a fixed amount of YFP-SUMO1, YFP-SUMO2 or YFP-SUMO3, were treated at different doses of As2O3 during 4 h and used for BRET assays. As previously demonstrated [40], the close proximity of several YFP moieties could cause quenching or interference phenomena that could lead to a decrease in the BRET signal. This explains that a stronger interaction is not observed with YFP-SUMO2 or -SUMO3 than with YFP-SUMO1 in response to As2O3. (TIF) [file pone.0044949.s001.tif]

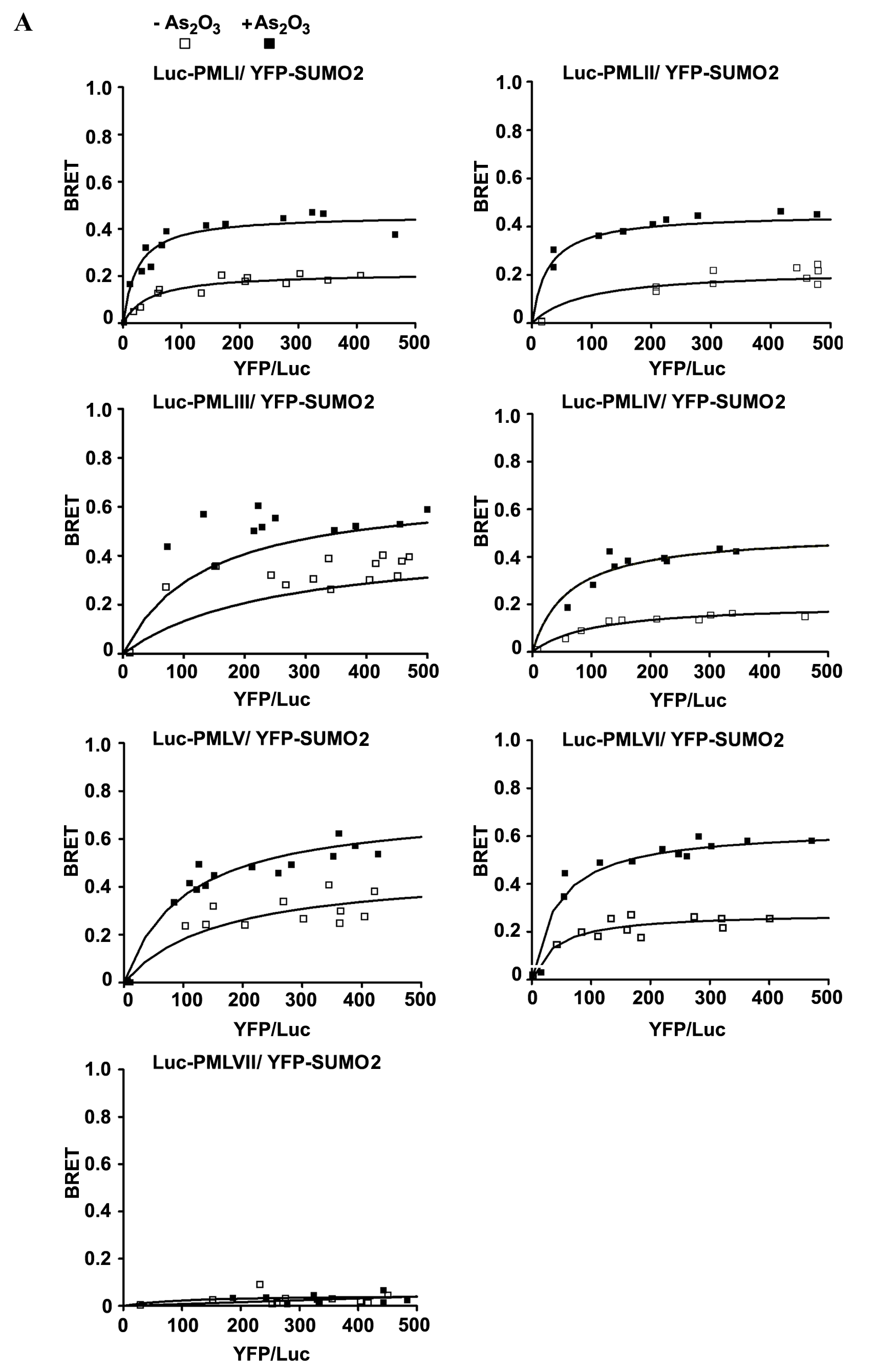

Supplement: Figure S2 — Detection by BRET of the increase in the SUMOylation of nuclear PML isoforms by SUMO2 or SUMO3 in response to As2O3 in living cells. HEK293T cells, transiently transfected with a fixed amount of a Luc-PML fusion (PML I to VII) and increasing amounts of YFP-SUMO2 (A) or YFP-SUMO3 (B), were treated in the presence or absence of As2O3 (5 µM, 4 h) and used for BRET titration assays. BRET saturation curves are presented for each PML isoform in the absence (open square) or presence of As2O3 (open square). (TIF) [file pone.0044949.s002.tif]

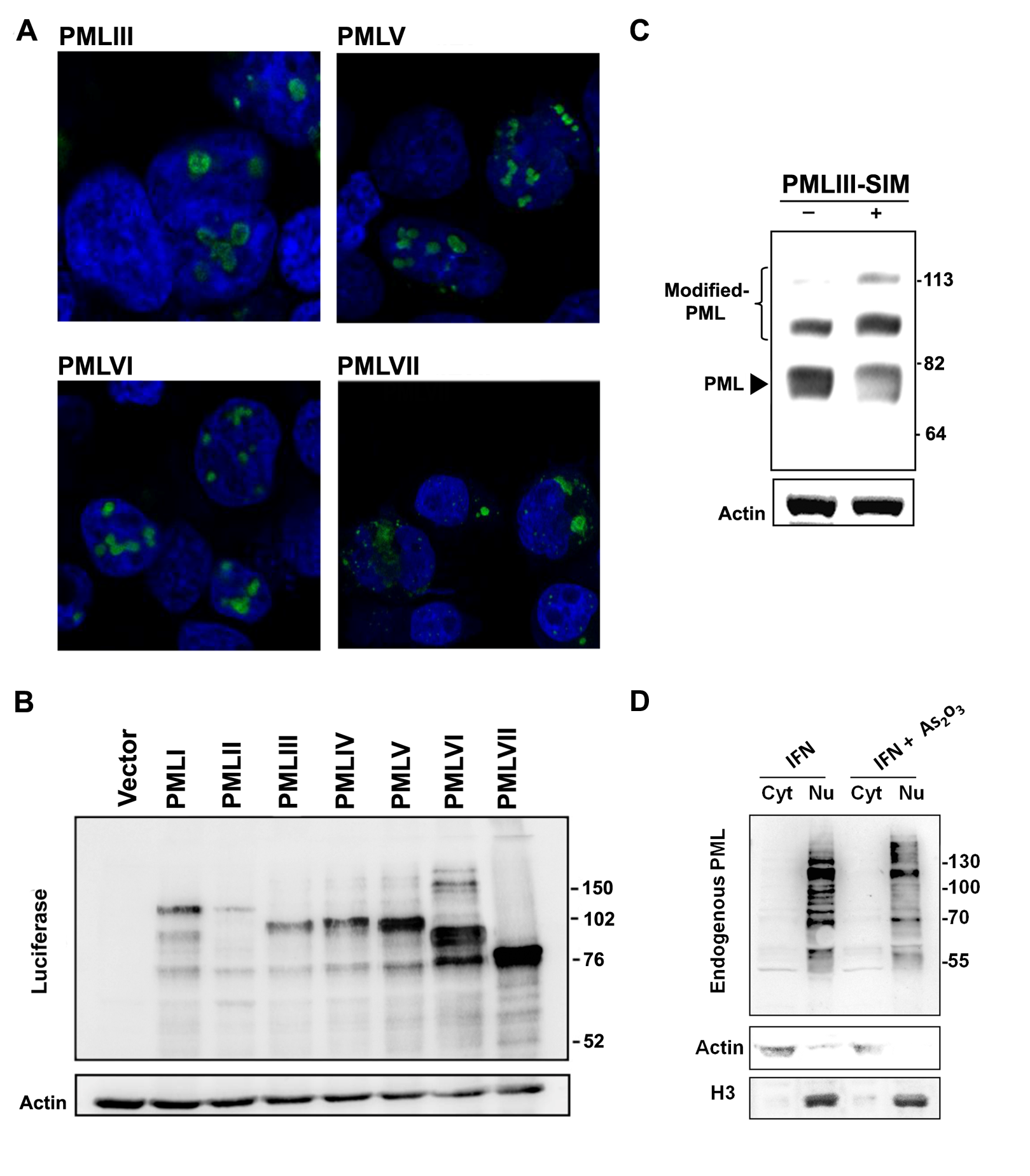

Supplement: Figure S3 — Immunofluorescence and Western blot analysis of PML isoforms. (A, B) Localization and expression of Luc-PML isoforms. HEK293T cells, transiently transfected with the empty vector or a Luc-PML fusion (PMLIII,V,VI or VII) were analyzed by confocal microscopy using anti-PML antibody (A) or by Western blot using anti-Luc or anti-actin antibodies (B). (C) The SIM of PML was not required for As2O3-induced PML SUMOylation. U373MG cells stably expressing PMLIII-SIM were treated with of 5 μM of As2O3 for 4 h. Total cell extracts were analyzed by Western blot for PML and actin expression. The unmodified PML isoforms are indicated by arrowheads and the modified PML species by brackets. (D) Analysis of cytoplasmic and nuclear extracts. U373MG cells prepared in duplicate were treated with 1000 units/ml of IFNγ. One day later As2O3 was added in one sample to a final concentration of 5 µM for 24 h. Cytoplasmic (Cyt) and nuclear (Nu) extracts were analyzed by Western blot with anti-PML, anti-actin and anti-histone H3 antibodies. (TIF) [file pone.0044949.s003.tif]

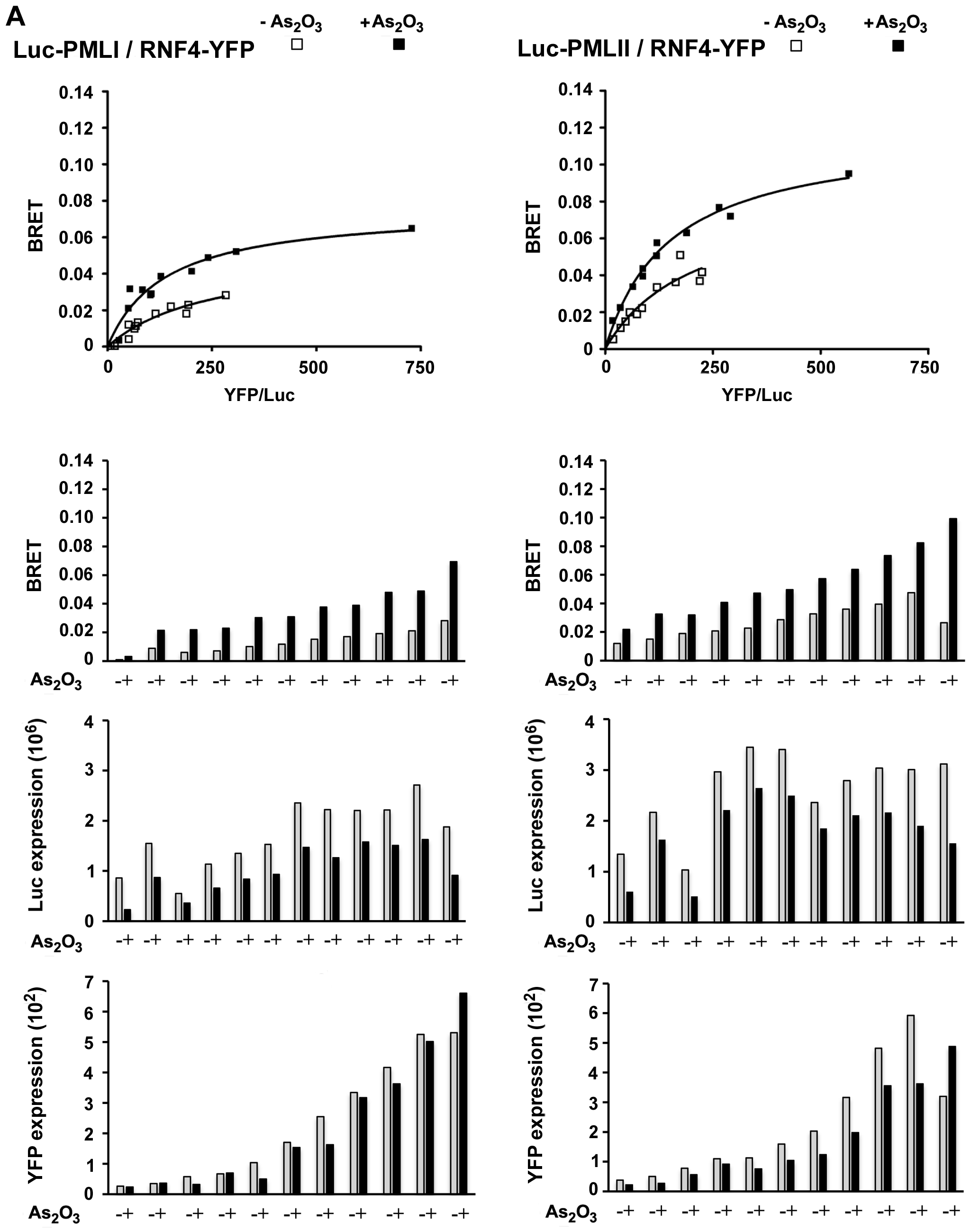

Supplement: Figure S4 — Interaction by BRET of RNF4 with SUMOylated PML isoforms (I, II, IV, V or VII). (A–C) HEK293T cells, transiently transfected with a Luc-PML fusion (I, II, IV, V or VII) and increasing amounts of RNF4-YFP, were treated in the presence or absence of with As2O3 (5 µM, 4 h) and used for BRET titration assays. BRET saturation curves in the absence (open square) or presence of As2O3 (closed square) are presented for each PML isoform in A) for PMLI and PMLII, B) for PMLIV and PMLV and C) for PMLVII. Bar graphs are for presenting the BRET signal (BRET), the luciferase expression and YFP expression of individual untreated (gray bars) and treated (black bars) samples; in the presence of RNF4-YFP, the expression of the Luc-PML fusion was decreased by the As2O3 treatment whereas the expression of RNF4-YFP stayed relatively constant. (TIF) [file pone.0044949.s004.tif]

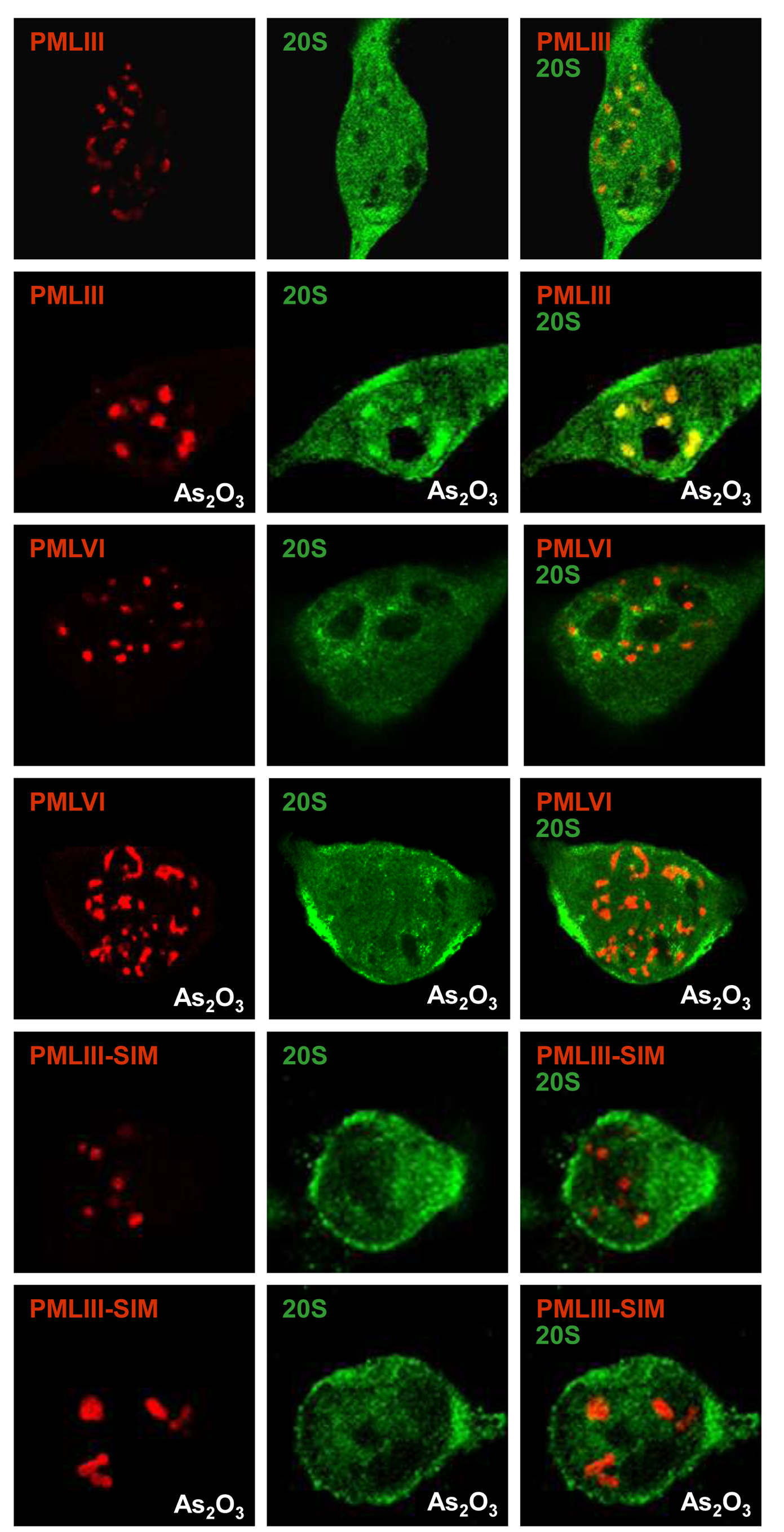

Supplement: Figure S5 — Requirement of the SIM for the recruitment of the 20S proteasome to PML NBs in response to As2O3. Confocal immunofluorescence analysis of PML and 20S core proteasome were performed on U373MG cells, stably expressing PMLIII, PMLIII-SIM or PMLVI, treated or not for 1 h with 5 μM of As2O3. PML and 20S core were detected with a mouse anti-PML and a rabbit anti-20S antibodies followed by the corresponding anti-IgG antibody conjugated to Alexa 594 (red) and 488 (green), respectively. As reported previously [5] colocalization between PMLIII and the 20S core is observed in some cells only in response to As2O3. The merged images revealed co-localization of PML and endogenous 20S core proteasome only for PMLIII in the presence of As2O3. (TIF) [file pone.0044949.s005.tif]
